# A Dynamic Fitting Strategy for Physiological Models: A Case Study of a Cardiorespiratory Model for the Simulation of Incremental Aerobic Exercise

**DOI:** 10.3390/jpm14040406

**Published:** 2024-04-11

**Authors:** Carlos A. Sarmiento, Alher M. Hernández, Miguel Á. Mañanas, Leidy Y. Serna

**Affiliations:** 1 Bioinstrumentation and Clinical Engineering Research Group, Bioengineering Department, Engineering Faculty, Universidad de Antioquia UdeA, Calle 70 # 52-51, Medellin 050016, Colombia; alher.hernandez@udea.edu.co; 2 Departament d’Enginyeria de Sistemes, Automàtica i Informàtica Industrial (ESAII), Universitat Politècnica de Catalunya, 08034 Barcelona, Spain; miguel.angel.mananas@upc.edu (M.Á.M.); leidy.yanet.serna@upc.edu (L.Y.S.); 3 Centro de Investigación Biomédica en Red de Bioingeniería, Biomateriales y Nanomedicina (CIBER-BBN), 28029 Madrid, Spain

**Keywords:** cardiorespiratory system, computer simulation, mathematical model, parameter estimation, predictive models

## Abstract

Using mathematical models of physiological systems in medicine has allowed for the development of diagnostic, treatment, and medical educational tools. However, their complexity restricts, in most cases, their application for predictive, preventive, and personalized purposes. Although there are strategies that reduce the complexity of applying models based on fitting techniques, most of them are focused on a single instant of time, neglecting the effect of the system’s temporal evolution. The objective of this research was to introduce a dynamic fitting strategy for physiological models with an extensive array of parameters and a constrained amount of experimental data. The proposed strategy focused on obtaining better predictions based on the temporal trends in the system’s parameters and being capable of predicting future states. The study utilized a cardiorespiratory model as a case study. Experimental data from a longitudinal study of healthy adult subjects undergoing aerobic exercise were used for fitting and validation. The model predictions obtained in a steady state using the proposed strategy and the traditional single-fit approach were compared. The most successful outcomes were primarily linked to the proposed strategy, exhibiting better overall results regarding accuracy and behavior than the traditional population fitting approach at a single instant in time. The results evidenced the usefulness of the dynamic fitting strategy, highlighting its use for predictive, preventive, and personalized applications.

## 1. Introduction

The increase in and variety of chronic diseases, consequences of a reactive medical approach, and the unsatisfactory results of generic diagnoses and treatments suggest the need for a predictive, preventive, and personalized medical paradigm [1,2,3]. This must be based on integrating healthcare personalization concepts, anticipation, risk assessment, and the determination of optimal treatment based on predictive tools [3,4]. Tools that predict a patient’s personalized health status and their risk of presenting pathologies based on their physiological information are preferable [5,6,7].

Mathematical modeling and computational simulation of physiological systems have proven to be support tools for medical science. Their applications have allowed the evaluation of clinical treatments, the development of training tools and medical education platforms, and the design of medical equipment [8,9,10,11,12]. These advancements are possible due to the models’ ability to predict clinically critical physiological variables under varying conditions and for different individuals and stimuli or influences. One of the key features of these modeling-based tools is the comprehensive portrayal of elements, functions, and interactions. This aspect sets them apart from other predictive tools, enabling a precise comprehension of intricate regulations and physiological processes [13,14].

Cardiorespiratory system models are relevant in clinical applications as they predict the variables influenced by complex regulatory processes, crucial for maintaining body homeostasis. This analysis is essential for diagnosing, monitoring, and treating diseases [15,16]. At present, there are several studies focused on verifying and using cardiorespiratory models in different situations, including exercise [17,18], cardiovascular surgeries [19,20], respiratory disorders [21,22,23], mechanical ventilatory support [24], and others. Among these situations, physical exercise can be especially important to diagnosis and clinical prediction because exercise is a natural stimulus that generates significant and well-characterized variations in related physiological systems [25,26].

Despite the advantages and variety of validated mathematical models of physiological systems, their usefulness in medical practice, particularly for predictive, preventive, and personalized approaches, is restricted by different factors. One of the most important is their mathematical complexity, which is more remarkable as their precision and biological relevance increase and is related to many parameters that are difficult to measure experimentally [13,14]. Although nominal parameter values have been provided in the literature for model simulations, these were derived under specific experimental conditions and subject characteristics. Using them for predictions gives a constrained and quite general idea of a physiological system’s dynamics, often neglecting the characteristics that vary among individuals and conditions [13].

The different fitting techniques reported so far allow for modeling personalization based on experimental data such as vital signs. These techniques are generally related to identifying parameters that are challenging to measure experimentally [14,27,28]. Even so, these methods primarily focus on recognizing constant characteristics, thereby limiting the utility of the fitted models. They are designed for the static prediction of physiological response, not accounting for alterations in model parameters due to lifestyle changes, aging, diseases, and so forth.

In our view, a much-needed application of physiological models as a predictive, preventive, and personalized medicine tool would involve a strategy to continuously optimize and customize their parameters as a function of time. Some studies involve continuously optimizing the model parameters to accommodate the effect of time on the change in systems’ behavior, potentially leading to favorable prediction outcomes for various applications [29,30,31]. This approach, known as time-varying systems modeling, has also been applied to models of physiological systems. However, its applications have mainly focused on small models that are described using only a few equations and for which the experimental data are directly related to predicting the interest variables [32]. Although its application could be extended to more complex models (involving different variables or complete self-regulation), its application ignores that the system’s responses may be attributed to the effects of other systems or controllers not directly related to the measured variables.

Our work presents a novel strategy for the dynamic fitting of physiological models with complete self-regulation. This approach enables the prediction of the future states of the modeled physiological system, even with a large number of parameters and limited experimental data for fitting. Our strategy uses techniques from previous works on fitting and modeling physiological systems, particularly focusing on a complex cardiorespiratory model tailored to predict healthy adult subjects’ responses to incremental aerobic exercise [33]. Using this model as a case study and challenged by dynamic aerobic exercise scenarios, we leverage experimental records from a longitudinal study to establish evaluation criteria both for a population and from personalized perspectives. Our findings suggest that our strategy significantly enhances the usability of complex mathematical models of physiological systems in predictive, preventive, and personalized contexts.

This paper is structured as follows. First, we present the dynamic fitting strategy. This includes identifying the parameter values at selected time points, modeling their temporal trend, and then validating their applicability in predicting the future states of the physiological system being modeled. Following this, the case study is introduced, detailing the cardiorespiratory model, experimental observations, simulations, and data analysis. Thirdly, we present the results of each procedure under two fitting scenarios, focusing specifically on validating our strategy. Finally, we discuss the outcomes of the proposed approach.

## 2. Materials and Methods

In this section, we introduce our proposed dynamic fitting strategy, which encompasses the time-specific fit of the physiological models, dynamic parameter modeling, and validation. Subsequently, we present a case study employing a comprehensive mathematical cardiorespiratory model and experimental data obtained from a longitudinal study.

### 2.1. Dynamic Fitting Strategy

The proposed dynamic fitting strategy involves static fitting of physiological models, along with dynamic modeling of the model parameters that most influence the system response. Based on observations from a longitudinal study, it aims to identify the model parameter values that minimize the differences between the physiological model predictions and the experimental data at specific instants of time. It involves three key procedures: (a) a time-specific fit of the physiological model incorporating sequential application of a static fitting strategy [28], (b) dynamic modeling of the model parameters to reflect their time trends, and (c) a strategy validation considering the future steady-state response of the modeled physiological system.

Given the natural variation in physiological systems over time due to biological degradation and various influencing factors, such as habits, events, and diseases [34,35,36,37,38,39,40], the proposed strategy leverages the longitudinal outcomes of the static fit to reveal the temporal changes in the physiological system’s characteristics. By modeling these time-related trends, future parameter values can be predicted, providing valuable insights into the system parameter dynamics. The following subsections explain in detail the procedures that are encompassed.

#### 2.1.1. Time-Specific Fit of Physiological Models

**Figure 1:**
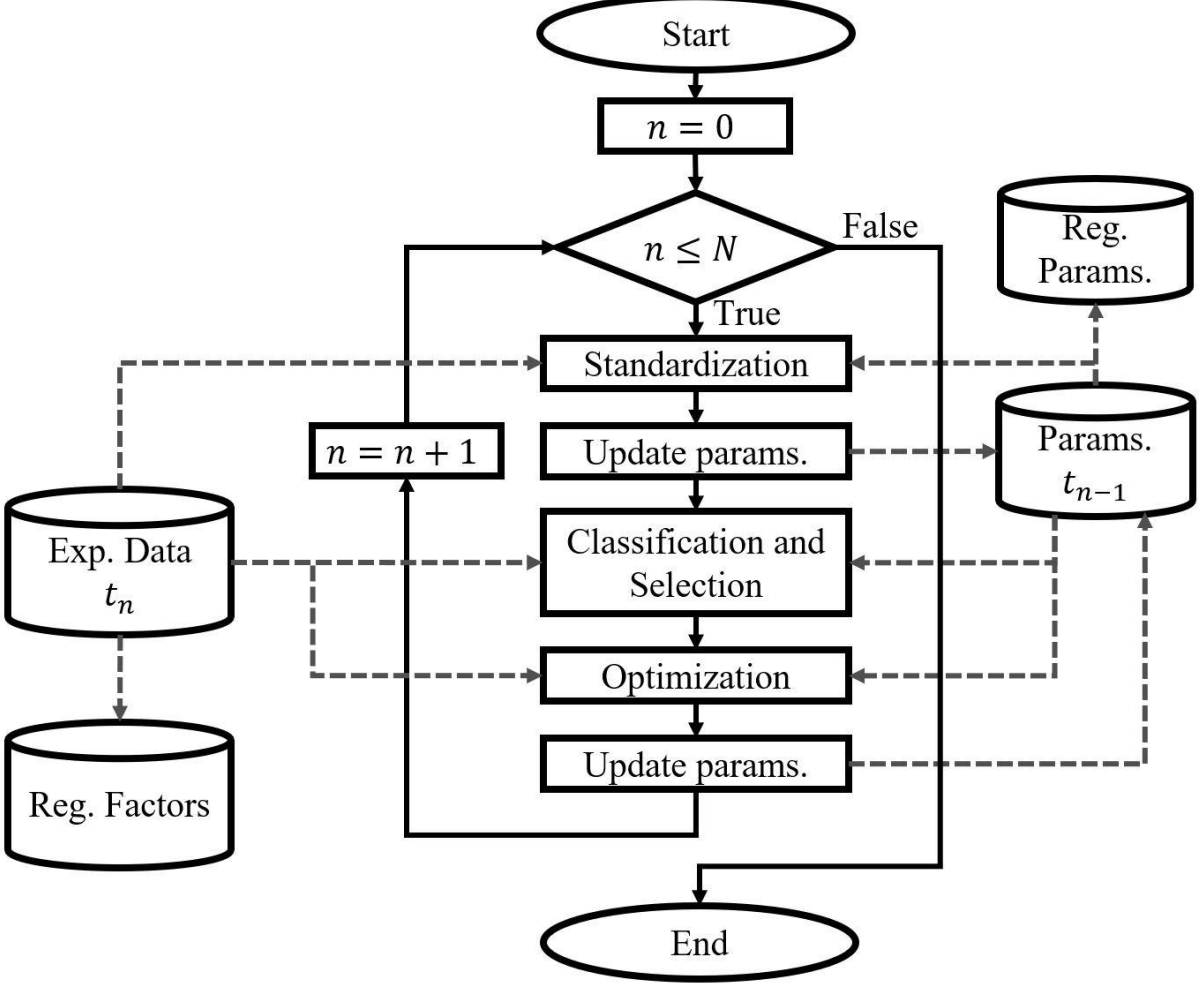
Flowchart for the time-specific fit procedure.

This procedure is focused on obtaining optimized model parameter values that best align the physiological model response with the experimental observations made at different instants of time during a longitudinal study. Figure 1 shows a general flowchart of the proposed fitting.

The number of results of the time-specific fit procedure will depend on the number of study samples or time instances (N) since each one (n) corresponds to a static fit of the model regarding the data recorded at each instant of time ($Exp.Data\;t_{n}$). The procedure comprises different sub-processes that must be sequentially applied over time, with the understanding that the results will reflect the temporal evolution of the physiological system for the same subject or population. The first fit (n=0) starts from the nominal values of the model parameters and the subsequent ones from the immediately preceding optimization result (Params. $t_{n-1}$). The sub-processes are applied iteratively, while the number of iterations does not exceed the number of instances in the experimental data (n<N). At each iteration, the parameter optimization results (Reg. Params.) and experimental information related to the factors that significantly influence the modeled system (Reg. Factors) are recorded. This information is useful for the subsequent dynamic parameter modeling procedure.

Physiological system models are highly intricate due to their many parameters. Attempting to fit them using a limited amount of experimental data can be computationally expensive and results in multiple optimization solutions that lack physiological meaning [14]. To address this issue, our time-specific fit procedure combines techniques such as standardization, classification, selection, and optimization of a reduced set of parameters. Consequently, it allows for predictions with adequate precision and accuracy for models considering many parameters and different stimuli and populations. The effectiveness of this procedure has already been tested in a case study involving a comprehensive cardiorespiratory model. Below, an overall explanation of it is provided. The reader can refer to [28] for more details.

**Standardization of simulation conditions**

Depicted as the first sub-process that generates results for “Update params.” in Figure 1, this involves adjusting the model parameters values based on the available experimental measurements. This aligns the model’s simulation conditions closer with those used as a reference for fitting, which ultimately reduces the computational costs by eliminating the need to optimize these parameters later. Standardized values are generally related to the characteristics of the subjects, the stimulus evaluated, or the environmental conditions. Parameters unaffected by standardization, which cannot be directly linked to the available experimental measures, retain the equivalents of their nominal values and are later considered in the classification and selection sub-procedures.

**Classification and Selection of Parameters**

This aims to reduce the complexity of the optimization problem by identifying a reduced set of the most representative parameters to be used during the model fitting [28]. It initially consists of classifying parameters based on their role, identifiability and sensitivity to variations, and relationship with the evaluated stimulus, as follows.

- Role: This consists of grouping the parameters into five sets according to the typical functions found in physiological models. These groups include time constants, conversion parameters, covariates, initial values, and gain and thresholds. The static fitting strategy focuses on adjusting the model predictions in a steady state, so only the sets of parameters with the role of gain and thresholds, whose variations have a physiological sense, are selected for applying the subsequent classification techniques.
- Identifiability and sensitivity to variations: This involves classifying the parameters selected by role according to how well conditioned they are to be identified [41] and ranks them according to their variation effect in the model predictions [14], employing both total and relative sensitivity analysis approaches. Their application involves improvements that allow evaluating different levels of stimulus and parameter values, while considering the error contribution regarding the experimental data [28]. As a result, two sets of parameters are identified. The first one corresponds to the parameters with the best results for subset selection and the highest total sensitivity. The second set comprises parameters with the best relative sensitivities for each variable, which, in turn, have a non-significant effect concerning the others.
- Stimulus: This encompasses the parameters that measure the impact of relevant mechanisms based on their classification by role.

Once the selection criteria are applied, the parameters eligible for optimization are identified. This process ensures that the optimization efforts are focused on the most relevant and impactful model parameters.

Parameter Optimization

This involves identifying the optimal values of the model parameters that reduce the difference between the model predictions and the experimental data [32]. The result is related to the second “Update params.” sub-process in Figure 1. This procedure focuses on solving an optimization problem, in which the outcome is associated with the choice and parametrization of an optimization algorithm, establishing the evaluation boundaries for the chosen parameters, and selecting a suitable metric for assessing the prediction capability of the model [27].

According to the referenced static fitting and validation strategy [28], three optimization stages are applied sequentially, related to the classification of and selection criteria for the parameters previously described. The first stage is the base fitting approach, which involves adjusting the identifiable parameters with the highest sensitivity. The second stage is the specific fitting approach, which focuses on reducing the prediction error of each variable without significantly affecting the result of the others by adjusting the parameters with the best relative sensitivity. The last stage is the stimulus-related fitting approach, which focuses on bringing the behavior of predictions closer to what is expected by modifying the related mechanisms.

#### 2.1.2. Dynamic Parameter Modeling

Its objective is to model the temporal trends in the parameters so that these trends can be used to predict the future fit of the physiological model. There is no specific structure defined for this modeling. Still, applying autoregressive techniques is recommended, considering their simplicity and the cumulative effect regarding time and other factors on physiological systems [34,35,36,37,38,39,40]. Therefore, the design of a MISO (Multiple-Input Single-Output) model is proposed for each parameter. This latter allows for predictions based on their past values and a temporal record of significant factors such as habits, events, or diseases.

**Figure 2:**
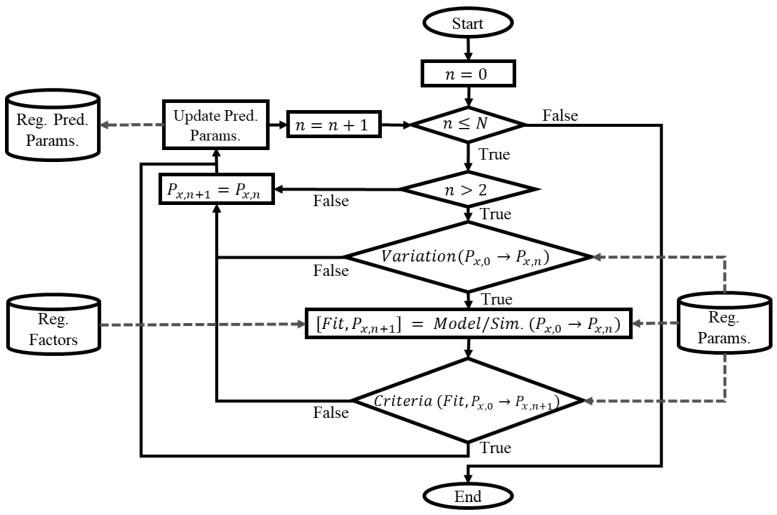
Flowchart for dynamic parameter modeling for dynamic fitting strategy.

Figure 2 shows the general flowchart for the dynamic modeling of a given parameter, denoted as $P_{x}$. This procedure focuses on predicting and storing the forthcoming value (Reg. Pred. Params.) at the next instant of time (n+1), based on the samples used for modeling within a range from 0 to n. Here, N denotes the total number of time instances available. The modeling and simulating process (marked as Model/Sim) involves only those parameters whose values are modified as a result of the time-specific fit procedure ($Variation\left(P_{x,0}\to P_{x,n}\right)$). Any parameter that remains unaltered retains its initial nominal or standardized value. Similar to the optimization process, it is essential to note that the predictions of the parameters could lack a physiological justification and should be evaluated before being accepted. The goodness-of-fit measures of the models and the rate of change in the parameter values can be used as criteria to define the use of the predictions or keeping the previous ones ($Criteria\left(Fit,P_{x,0}\to P_{x,n}\right)$).

#### 2.1.3. Validation

This procedure focuses on evaluating the results yielded by the proposed strategy based on its ability to accurately predict the fitting of the physiological model at a future point in time. For this purpose, the experimental values of the variables at each instant are compared against their respective predictions using the physiological model using three sets of parameter values: (a) parameters that are specifically fitted to the experimental data at each selected time point, which represent a time-specific fit and are the gold standard of the optimization process, (b) parameters resulting from the traditional single-time fit approach, which consist of the fitting results for the first instant of time using the experimental results of the entire population, and (c) the predicted parameters values obtained using the proposed dynamic fit strategy by dynamically modeling the selected model parameters.

Given that the sequentially implemented static fitting strategy does not involve responses to transient changes, the precision, accuracy, and directional aspects of the physiological model’s predictions are evaluated only when the steady state of the variables is reached. This process involves selecting and using a metric that allows for an assessment of the model’s performance, usually associated with calculating the prediction error.

### 2.2. Case Study

#### 2.2.1. Cardiorespiratory Model

**Figure 3:**
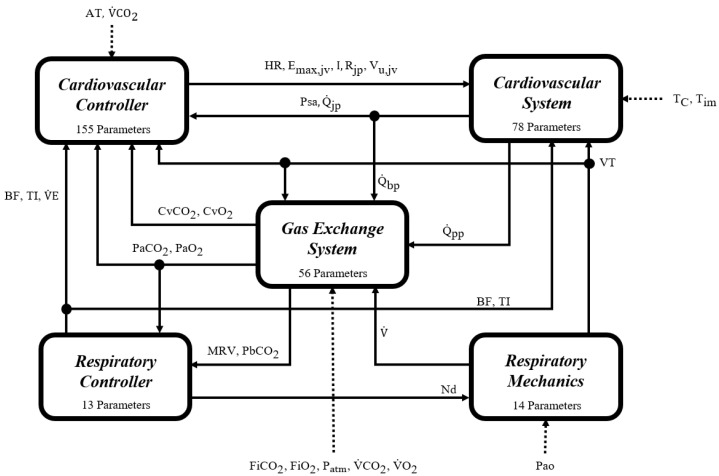
Schematic representation of the CR model. This diagram illustrates the overall structure of the case study model, showcasing the integrated systems along with their respective controllers. See Table 1 for symbol descriptions. The mathematical equations delineating the functionalities of all systems and controllers are detailed in the Supplementary Materials and in the model-building paper [33].

The mathematical cardiorespiratory model (CR model) used as a case study is a multi-compartmental self-regulated model of the cardiorespiratory system. It comprises 316 parameters and 170 equations distributed across different integrated mechanisms to represent the cardiovascular circulation system, the respiratory mechanic system, the gas exchange system, and the respiratory and cardiovascular controllers [33]. It is the result of adapting previous models reported of systems, controllers, and mechanisms. It has been fitted and validated using experimental data from healthy adult individuals during rest and progressive aerobic exercise [28]. Figure 3 delineates a schematic representation of the CR model, while Table 1 provides detailed descriptions of the symbols utilized within the model diagram. These references constitute essential instruments for facilitating a deep and comprehensive exploration of the model’s framework.

The cardiovascular portion of the CR model encompasses a detailed representation of the heart and the circulatory system, including both pulmonary and systemic circulation. Its sophistication lies in the incorporation of various physiological aspects, such as the dynamics of blood flow through different valves and vascular beds. Notably, it incorporates modifications to accurately simulate moderate aerobic exercise, detailing the effects of muscle contractions and respiration on venous return.

The cardiovascular controller focuses on facilitating reflex control through a complex network of neural pathways and effector mechanisms. It adeptly responds to a range of stimuli, including changes in oxygen and carbon dioxide levels, thereby modulating cardiovascular responses during aerobic exercise.

The respiratory module of the CR model outlines the mechanics of the pulmonary and upper airway systems. This model segment has been enhanced to simulate the intricacies of muscle pressure waves and air movement during the respiratory cycle, effectively representing both the inspiratory and expiratory phases.

The gas exchange system provides a comprehensive depiction of the mechanisms governing the interaction between the cardiovascular system and respiratory mechanics, focusing on the regulation of blood gas concentrations. This system intricately details the processes involved in gas exchange, both at the cerebral and tissue levels, incorporating essential mechanisms that are particularly significant during exercise.

The respiratory controller in the CR1 model integrates the central and peripheral chemoreceptors and a metabolically related neural drive component. This setup, which utilizes inputs from the gas exchange system, facilitates precise estimation of the ventilatory demand, maintaining optimal arterial blood gas levels during physical activity. Additionally, it employs an optimization approach to adjust the breathing pattern each cycle, minimizing the work of breathing (WOB) and fine-tuning variables such as VT, BF, and inspiratory and expiratory times (TI and TE), thus ensuring nuanced regulation of respiratory control during exercise.

For a more in-depth understanding and exploration of the CR1 model, readers are encouraged to refer to the Supplementary Materials. This material provides a comprehensive insight into the model, facilitating a deeper comprehension of its complexity and functionalities.

#### 2.2.2. Experimental Data

The experimental data used to evaluate the strategy corresponded to signals and measurements from cardiorespiratory variables recorded in an observational and longitudinal study. This was based on an incremental, submaximal, and multistate cardiopulmonary exercise test performed using a programmable cycle ergometer under controlled environmental conditions. The test lasted for a total of 45 min, segmented into five distinct phases: rest (2 min), warm-up (4 min), active exercise (25 min), recovery phase (9 min), and concluding with a final resting period (5 min). During the exercise stage, five consecutive steps were carried out with load increments of 25 W every five minutes and at a constant pedaling speed of 60 RPM.

The recorded information was divided into two databases to analyze the usefulness of the dynamic fitting strategy from population-wide and personalized perspectives. The first database (DB1) corresponds to the longitudinal record of twenty-seven male adult volunteers at three different instants with more than six months of difference between them. The second database (DB2) corresponds to the records of three male adult volunteers matched by degree of consanguinity, medical history, and lifestyle but with different ages, which are proposed as an approximation of the longitudinal records of one same subject at six different stages of his life, consistent with the approach of stratified medicine [42,43]. These subjects are male members of the same family, corresponding to a father and his two sons who live together and have similar habits. The first two records correspond to the youngest subject, the next three to his brother, and the last to his father.

All the participants registered during the study were deemed healthy, encompassing individuals with diverse physical fitness levels, from sedentary to those regularly engaging in physical training. They were non-smokers and had no history or present symptoms of cardiovascular, pulmonary, metabolic, or neurological disorders. Additionally, none had pacemakers or any other types of implanted electrical stimulators. The records were composed of the signals of $\dot{V}E$, TI, VT, BF, HR, oxygen uptake ($\dot{V}O_{2}$), carbon dioxide output ($\dot{V}{CO}_{2}$), alveolar oxygen partial pressure ($PAO_{2}$) and alveolar carbon dioxide partial pressure ($PA{CO}_{2}$) and measurements of systolic blood pressure (PS), diastolic blood pressure (PD), and mean arterial blood pressure (PM). The records also comprised characteristics of the environment, such as $P_{atm}$, ${FiCO}_{2}$, and ${FiO}_{2}$.

Quantifiable information related to factors other than age reported significantly influencing the cardiorespiratory system was also recorded. These data accounted for habits such as diet [35,36], physical activity [38,39], and quality of sleep [37,44], related to measures of body mass index, average hours of weekly physical activity, and average hours of daily sleep, respectively. Other relevant factors, such as accidents or illnesses, habits such as smoking, or family history of diseases, were not reported, considering that they were regarded as exclusion criteria. Medication use was only reported by the oldest subject of the DB2 and was related to blood pressure control. Table 2 presents the information recorded for each database related to the environmental conditions, the characteristics of the volunteers, and the factors mentioned above. The data shown for DB1 correspond to the mean and standard deviation of the measurements.

All the methods received authorization from the Ethical Committee for Human Research of the University Research Department (SIU) of the University of Antioquia (approval certificates 16-59-711, 17-59-711, and 18-59-711). These procedures adhered to the standards set by the Declaration of Helsinki. Written agreements were secured for every participant following a briefing on the experiment’s procedure and the associated risks.

#### 2.2.3. Computational Implementation and Data Analysis

The measured values of $\dot{V}O_{2}$ and $\dot{V}{CO}_{2}$ served as the model inputs to simulate exercise intensity levels. This method has been previously employed in simulating aerobic exercise within physiological models [20,45]. The rationale behind this approach is that the physiological response to such a stimulus is intrinsically linked to the metabolic rates of $O_{2}$ uptake and ${CO}_{2}$ production, which are directly affected by the workload during exercise (the physiological model does not consider the workload as a model input).

The average of each measurement during the last minute for each subject (except for PS, PM, and PD) was used as a representative sample of each phase of the exercise test under steady-state conditions. The experimental data were constrained using $\dot{V}{CO}_{2}$ as a reference, starting at 0.3 L/min, which corresponds to the resting state and consists of the minimum value observed across all the subjects’ records, and ending at the respective calculated value of AT because the model was defined only for aerobic exercise.

Each temporal fitting of the model parameter values references the parameter values from the most recent optimization result, except for the first fit of each database. In this case, the nominal values reported in [33] are used, reflecting a traditional single-time fit approach.

Each stimulus level was simulated for 2000 s to guarantee steady-state conditions for all the evaluated variables, as reported in [33]. The model’s steady-state responses were determined by calculating the mean values of each variable during the final minute of each simulated stimulus level.

The model simulations, data processing, and statistical tests were performed using SIMULINK/MATLAB^®^ version R2023a. The computational specifics of the simulation were aligned with those documented for the model’s validation, utilizing the numerical solver ODE23 (Bogacki and Shampine BS23 algorithm for the solution of ordinary non-rigid differential equations) and a variable step size between $1\times 10^{-2}$ and $1\times 10^{-3}$ seconds. The simulations were conducted only once, as all the model equations are deterministic. Below, the specific implementation details for each procedure are outlined.

**Time-Specific Fitting of the Physiological Model**

*Standardization of the simulation conditions*

The parameters of the physiological model that were assigned according to the experimental data included $FiO_{2}$, $Fi{CO}_{2}$, $P_{atm}$, basal respiratory tidal volume (${VT}_{n}$), AT, total blood volume ($V_{tot}$), and constant values of unstressed blood volume. $FiO_{2}$, $Fi{CO}_{2}$, and $P_{atm}$, accounting for the environmental conditions, were considered constant in all the records and equal to 21.0379%, 0.0421%, and 640 mmHg, respectively. The values of ${VT}_{n}$, AT, $V_{tot}$, and unstressed blood volume are associated with the distinct characteristics of the subjects. The value of ${VT}_{n}$ was determined from the VT measurements; AT was calculated using the noninvasive technique v-slope [23,46]. $V_{tot}$ in ml was also estimated for each record in relation to the body surface area (BSA) in $m^{2}$ according to Equations (1) and (2) [47]. Finally, the parameters related to the unstressed blood volumes were calculated using the proportion of their reported nominal values regarding $V_{tot}$ [33].

(1) $$BSA=\sqrt{\frac{\left(Weigth\cdot High\right)}{3600}},$$

(2) $$V_{tot}=1000\left(3.29\cdot BSA-1.29\right),$$

The values of ${VT}_{n}$, AT, and $V_{tot}$ used for the standardization of each fit are presented in Table 3. The data presented for DB1 correspond to the mean and standard deviation, except for AT, whose value corresponds to the median and interquartile range considering the data distribution.

*Classification and Selection of Parameters*

Classification and selection of the parameters were performed based on the steady-state simulation results by applying changes in stimulus level and parameter value variations. Identical stimulus levels and parameter variations as those reported for the static fitting strategy previously applied to the same model were used [28]. Three exercise stimulus levels were simulated using $\dot{V}O_{2}$ and $\dot{V}{CO}_{2}$ as the model inputs, corresponding to states of rest, an intermediate level of exercise, and AT. The identifiability and sensitivity of the model parameters selected by role (gain and thresholds) were evaluated throughout five uniformly distributed percentage variations in a range of ±5% of the reference value. The number of parameters selected was the same as that reported for the model according to the static fitting strategy.

The fitting approach related to the stimulus was not applied because it did not significantly decrease the prediction error, and the exercise mechanisms had already been fitted to the model [28].

*Parameter Optimization*

The optimization algorithm implemented corresponds to the Covariance Matrix and Adaptation Evolution Strategy (CMA-ES). This stochastic global optimization algorithm is grounded in adaptive and evolutionary principles [48,49]. Its selection was based on its favorable outcomes in terms of the convergence speed, precision, and accuracy when fitting the case study model [28] and other related ones [27]. The parameter values applied in the implementation of the CMA-ES algorithm align with those reported for the model according to the static fitting strategy [28].

The evaluation ranges for the parameter optimizations were defined in accordance with the static fitting strategy [28]. The parameter values reported as nominal for the model were used as the variation references [33]. A general evaluation range of ±30% regarding the nominal value was established and expanded to ±50% for weighting parameters unrelated to direct physiological measures. These ranges were modified according to the information reported about the optimization results or physiological sense (values with physiological justification).

Steady-state model predictions of $\dot{V}E$, TI, VT, BF, HR, $PAO_{2}$, $PA{CO}_{2}$, PS, PD, and PM for eight consecutive, equidistant, and incremental step values of $\dot{V}O_{2}$ and $\dot{V}{CO}_{2}$, spanning from rest to AT as the experimental data, were used for the parameter optimization. The cost function (CF) employed corresponds to Equation (3), utilizing the same root mean squared error (RMSE)-based metric as applied in the reported fitting of the case study model [28].

(3) $$CF=\frac{1}{I}\sum_{i=1}^{I}\sqrt{\frac{1}{K}\sum_{k=1}^{K}{\left(\frac{y_{{exp}_{i,k}}-y_{{sim}_{i,k}}}{y_{{exp}_{i,k}}}\right)}^{2}}$$

where $y_{exp}$ and $y_{sim}$ correspond to the experimental and simulated values of the variable, respectively; I and K represent the number of variables and stimuli levels; and the subscripts i and k designate each specific variable and stimulus level.

**Dynamic Parameter Modeling**

To effectively model a dynamic parameter, it is essential that the parameter has been optimized at least twice at two consecutive instants of time. Thus, for DB1, only the optimized parameter values of record three were predicted, while for DB2, the predictions encompassed the optimized parameter values from records three to six. Specifically, only the parameters demonstrating significant variations in their value during the optimization procedure were modeled as dynamic parameters. The remaining parameters retained their previous values, whether they were nominal, standardized, or previously optimized.

Deterministic and parametric MISO models were implemented for each dynamic parameter. The implemented model structure corresponded to an ARX model (autoregressive with exogenous input), selected to consider the effect of the past values of the outputs and inputs on the current value [32]. Equation (4) corresponds to a representation of the model structure.

(4) $$A\left(q\right)P\left(n\right)=\sum_{i=1}^{I}B_{i}\left(q\right)F_{i}\left(n-k_{i}\right)+e\left(n\right),$$

where P represents the parameter’s value at each instant of time; $F_{i}$ is the ith input, which is associated with the factors evaluated; A and B are polynomials expressed in the time-shift operator $q^{-1}$ and are related to the parameter and the inputs, respectively; I is the total number of inputs; $k_{i}$ is the input delay; and $e\left(n\right)$ is considered random noise.

For each model, the following inputs were used: the time elapsed between records, the average weekly physical activity, the average daily sleep hours (Table 2), and AT (Table 3). To calculate the time, the difference in age between each record was accumulated, and a value of zero for the first record was assigned. AT values have been used because they account for the metabolic response of the subject to cardiorespiratory changes [46,50]. Except for time, the values of the previous record’s inputs were considered equivalent to the values in the record to be predicted.

Different model orders associated with the polynomials were evaluated for each parameter, and the model with the best fit was selected. The past values of the parameters and the inputs were interpolated, considering that the design of the models requires constant sampling times. Sampling times between 0.1 and 0.4 years were evaluated in this work, considering the temporal difference between records for each fit. The orders of polynomials A and B were evaluated from zero to the maximum number of samples available. $k_{i}$, the input delay, was considered zero regarding all the inputs due to the limited number of available records. The coefficients of the polynomials for each model were estimated using the least-squares method. The best-order selection was based on applying Akaike’s Information Criterion (AIC) according to Equation (5).

(5) $$AIC=ln\left(V\right)+\frac{2d}{N},$$

where V is the loss function (normalized sum of squared prediction errors), d is the total number of parameters in the structure in question, and N is the number of data points used for the estimation.

Criteria based on the goodness of fit of the models and the variation in the predicted value regarding a bounded range were applied to evaluate the physiological justification for the predictions. Models with a goodness of fit of less than 60%, sign changes, or variations greater than 50% regarding the range of recorded values were discarded, and the values corresponding to the immediately previous record were used instead.

**Validation**

Validation consisted of comparing the simulation results of the case study model using (1) optimized parameter values for each record (time-specific fit approach), (2) nominal parameter values (single-time fit approach), and (3) predicted parameter values from the identified dynamic parameter models (dynamic parameter fit approach). These comparisons aimed to demonstrate the usefulness of the dynamic fitting strategy from the perspective of predicting the future state of the modeled system against the traditional approach of using the traditional population fit results at a single instant of time.

*Prediction Error*

For each approach, the effectiveness of the model performance was assessed by calculating the prediction error (PE) in relation to the steady-state experimental measures of the recorded cardiorespiratory variables ($\dot{V}E$, TI, VT, BF, HR, $PAO_{2}$, $PA{CO}_{2}$, PS, PD, and PM) for eight incremental step values of $\dot{V}O_{2}$ and $\dot{V}{CO}_{2}$ from rest to AT, as in the optimization process. The metric used to calculate the PE corresponds to Equation (6), a modification of the Mean Absolute Error (MAE) that allows an interpretation of the differences as a proportion of the experimental data. It considers the error for each subject, variable, and level of stimulus as follows:

(6) $$PE\left(\%\right)=100\cdot \frac{1}{N}\sum_{j=1}^{N}\left({Median}_{i,k}\left(\left|\frac{y_{{exp}_{i,j,k}}-y_{{sim}_{i,j,k}}}{y_{{exp}_{i,j,k}}}\right|\right)\right)$$

where $y_{exp}$ is the experimental variable value; $y_{sim}$ is the simulation prediction value; N denotes the number of variables; and the subscripts i, j, and k are indexes that represent each subject, variable, and stimulus level, respectively.

*Statistical Analysis*

The Wilcoxon signed-rank statistical test was applied to evaluate whether the distributions of the PE results of the used fitting approaches were different. As a non-parametric test, the Wilcoxon signed-rank test is widely recommended for two populations when observations are paired [51]. The test was applied between the PE results for all pairs of different approaches. The results were considered statistically significant for ρ values less than 0.01 (strong differences) and less than 0.05 (mild differences). ρ value results greater than 0.05 were not considered statistically different.

## 3. Results

### 3.1. Time-Specific Fitting of the Physiological Model

Table 4 shows the PE results for the model fitted to each record of the databases. The results regarding the variables correspond to the median and the interquartile range.

For both databases, it is observed that the highest PE values are generally related to the respiratory variables and the lowest to the gas exchange variables. More specifically, DB2’s fittings generally show results with lower variabilities and lower overall PEs. It is also highlighted that the highest values of overall PE in DB2 are related to the most significant changes in the cardiorespiratory system according to the proposed approach (Section 2.2.2. Experimental Data).

### 3.2. Dynamic Parameter Modeling

Table 5 and Table 6 compare the parameter predictions obtained via the proposed dynamic fit approach (Pred.) with the optimized results (Fitted) from the time-specific fit approach for the records evaluated of each database. The results for each parameter are grouped according to the directly related system or controller. A definition and description of each parameter are omitted in this section for the sake of being concise. Readers seeking comprehensive details are directed to the publication on the referenced case study’s model [33]. The symbol (*) indicates that the parameter was not predicted for the record because there was no modification regarding the previous values, but it was optimized for the evaluated record. The symbol (**) indicates that the predicted value did not satisfy the physiological justification criteria and therefore the value from the previous record was used instead.

Most of the parameters optimized for DB1 show slight differences regarding the predicted ones and those that were not modified (*) (variations of less than 10%). Therefore, similar simulation results could be expected. In this case, all the predictions were in accordance with the defined physiological justification criteria, which suggests good fits for a restricted number of samples and predictions with consistent variations considering the prediction time and the input data.

From the results related to DB2, it was observed that the number of non-modeled parameters (*) decreased with the number of samples, which is related to the greater coverage of the prediction of physiological characteristics related to cardiorespiratory variations. Even so, the number of predictions that did not satisfy the physiological justification criteria increased for the last two records (**), related to lower goodness-of-fit measures and more significant variations, mainly for record six, considering the longer prediction time (Table 2). The most significant differences were obtained for the respiratory controller parameters due to the results of the time-specific fit for each record. They could be related to the higher reported errors of the physiological model regarding the respiratory variables [28].

### 3.3. Validation

The validation results correspond to a comparison of the model predictions in a steady state with the experimental data and the PE values at each evaluated instant of time. The compared simulations correspond to those obtained using the parameters resulting from the dynamic fit approach (dynamic fit) against those of the traditional single-time fit approach (single-time fit). The time-specific fit results for each database record are presented as a reference (time-specific fit). Additionally, the PE results are detailed by presenting the overall value, mean, median, interquartile range, and any statistically significant differences among predictions.

**Figure 4:**
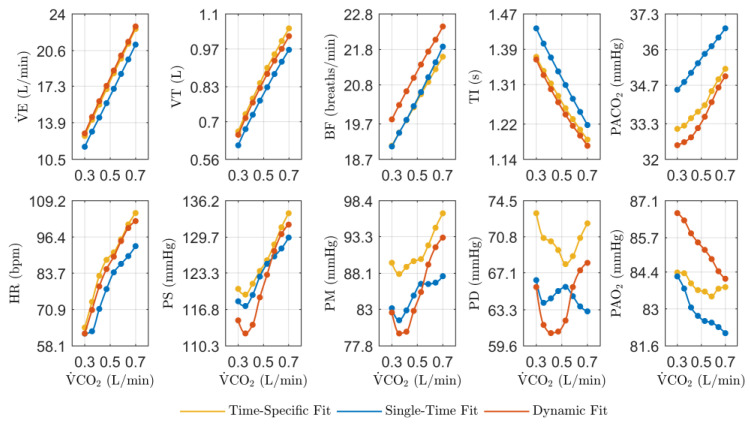
Steady-state simulation results against experimental data from the third DB1 record in function of experimental levels of exercise ($\dot{V}CO_{2}$). Simulations were based on the third DB1 record following three approaches: time-specific fit, single-fit, and proposed dynamic fit. They aimed to evaluate the physiological model performance from a population perspective.

Figure 4 shows the steady-state results of the model predictions for the evaluated physiological variables in function of the experimental levels of exercise ($\dot{V}CO_{2}$) regarding the third DB1 record. It compares the model simulations for the three approaches: the time-specific fit, the single-time fit, and the proposed dynamic fit.

It was shown that the simulations of the dynamic fit approach were the most similar to those of the time-specific fit approach for this record, mainly for the variables $\dot{V}E$, VT, TI, $PACO_{2}$, and HR, both in terms of magnitude and behavior. Although PM and PD show some of the most significant differences in magnitude, their behaviors concerning the stimulus are similar, mainly for PD, compared to the results of the traditional single-time fit approach. Some variables that present the most significant differences for the dynamic fit approach are related to the parameter values with the highest variations regarding those optimized for the record (Pred. and Fitted values for record 3 in Table 5, respectively). Thus, the results of PM and PD can be attributed mainly to the values of $I_{0,met}$ and ${PaCO}_{2,n}$ and BF to the values of $\lambda_{2}$ and $P_{max}$. For $PAO_{2}$, the differences in the parameter values are not so significant, so the results may mainly be related to the effect of the systemic arterial pressures on the blood flows that enter the gas exchange system (Equations of Gas Exchange and Mixing and Gas transport in the Supplementary Materials).

**Figure 5:**
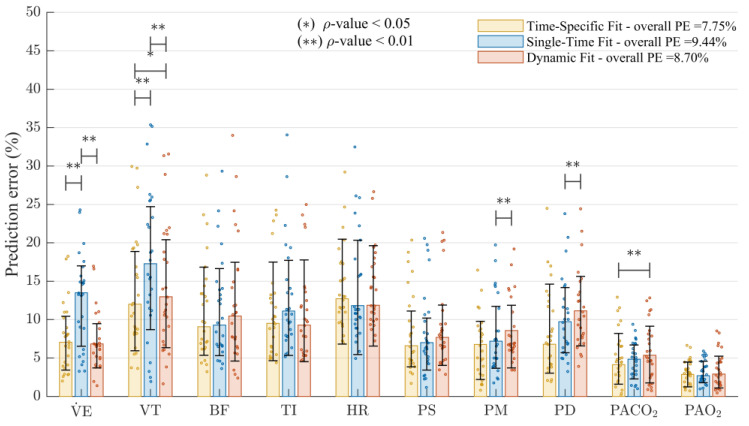
Percentage prediction error (PE) obtained for the physiological model responses regarding the third DB1 record following three approaches: time-specific fit, single-time fit, and proposed dynamic fit. The height of the bars shows the median values, and the error bars indicate the interquartile range (values between 25 and 75% of the data distribution). Statistically significant differences between the PE values of each approach were found using the Wilcoxon signed-rank test and are shown as (*) for ρ < 0.05 and (**) for ρ < 0.01.

Figure 5 shows the PE values obtained for the physiological model predictions regarding the third DB1 record. The data are presented as the median and interquartile range of the PE results for each approach. The average PE values for each subject are presented as individual points. The overall PE values and statistically significant differences between the results for each approach are also presented.

The obtained overall results indicate that the simulations related to the dynamic approach are more suitable than those of the traditional single-time fit approach. The highest errors associated with the traditional single-time fit approach are for $\dot{V}E$ and VT, for which the most statistically significant differences are obtained regarding the time-specific fit results. It is also evidenced that the most significant PE contribution to the dynamic fit approach is mainly related to the variables PM and PD.

**Figure 6:**
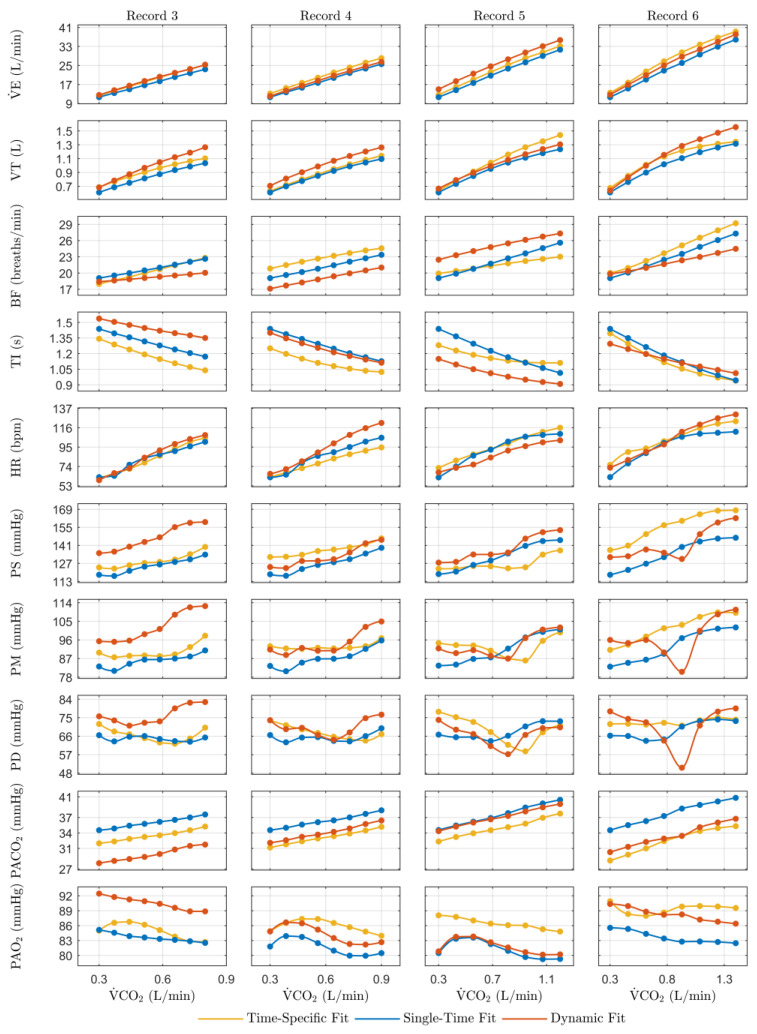
Steady-state simulation results for the evaluated physiological variables in function of experimental levels of exercise ($\dot{V}CO_{2}$). Simulations were based on the third to the sixth DB2 record following three approaches: time-specific fit, single-fit, and proposed dynamic fit. They aimed to evaluate the physiological model performance from a personalized perspective. The results are presented in a matrix such that the rows correspond to each evaluated variable and the columns to the compared records.

Figure 6 shows the steady-state results of the model predictions for the evaluated physiological variables in function of the experimental levels of exercise ($\dot{V}CO_{2}$) from the third to the sixth DB2 records. It compares the model simulations for the three approaches: the time-specific fit, single-time fit, and proposed dynamic fit. The results obtained for each record are described below.

The simulations of the dynamic fit approach related to the third DB2 record show less similarity to the time-specific fit results than for DB1, although they match the same record number. The most significant differences are presented for the variables BF, TI, PS, PM, PD, $PACO_{2}$, and $PAO_{2}$, and are related to the parameter values used for the simulations (Table 6). The value of $\lambda_{2}$, a weighting factor relating to the work of breathing during expiration, significantly affects the regulation of BF and TI (Equations of the Breathing Pattern Optimizer in the Supplementary Materials). The lower magnitudes of the systemic arterial pressures are related to a lower predicted value of ${KE}_{lv}$ due to its effect on the left ventricular pressure signal (Equations of The Heart in the Supplementary Materials). For $PACO_{2}$ and $PAO_{2}$, although the differences could also be related to the parameter values, especially $K_{2}$ for $PACO_{2}$, considering the high sensitivity of the gas exchange system to cardiovascular feedback (Equations of the Gas Exchange and Mixing in the Supplementary Materials), it could be mainly attributed to the lower systemic blood pressures.

Regarding the fourth DB2 record, the dynamic fit approach results showed improvements in the behavior and magnitude of the variables compared to the previous ones, mainly for TI, $PACO_{2}$, and $PAO_{2}$. The difference demonstrated for the respiratory variables could be related to the high variation between the predicted parameters, obtained using the dynamic fit approach, and the optimized parameters, obtained using the time-specific fit approach, regarding the respiratory controller (Table 6, Pred. and Fitted values in record 4, respectively). The difference between the HR slopes regarding the stimulus is mainly due to the prediction of higher sympathetic activity ($G_{T,s}$) and lower parasympathetic activity ($G_{T,v}$) (Equations of the Effectors for Reflex Control in the Supplementary Materials). This result is coherent considering the time trend in the previous records of these parameters. The improvement in the simulations of systemic blood pressure is due to the increased similarity between the predicted and optimized values of ${KE}_{lv}$. Regarding $PACO_{2}$ and $PAO_{2}$, considering that the differences in the related parameters are insignificant, the improvement can be attributed mainly to the best-predicted systemic blood pressure values.

As shown in Figure 6, the results from the dynamic fit approach for the fifth DB2 record show more similar behaviors to those from the time-specific fit, mainly for the variables BF, TI, PS, PM, and PD. This fact can be attributed to the minor difference between the predicted (dynamic fit) and optimized (time-specific fit) parameter values compared to previous records due to better predictions and the use of values of earlier optimizations that are not modified for the last fit (Table 6, Pred. and Fitted values in record 5, respectively). Regarding the differences in magnitude between the dynamic fit and the time-specific fit results, a higher $\dot{V}E$ value could be mainly related to a high dead space volume (${V0}_{dead}$) (Equations of the Ventilation Controller in the Supplementary Materials); the lower TI and the higher BF could be related to a higher $P_{max}$ value [45]; a lower HR could be due to a higher $T_{0}$ (Equations of the Effectors for Reflex Control in the Supplementary Materials); and the differences in $PACO_{2}$ and $PAO_{2}$ could be attributed to the variation in the concentration of hemoglobin ($C_{1}$) and the difference in the blood and respiratory flows (related to $\dot{V}E$ and systemic arterial pressure).

The dynamic fit approach results for the sixth DB2 record present magnitudes more similar to those of the time-specific fit approach than those of the traditional single-time fit approach. Regarding the behavior, there was an increase in the variation regarding the results for previous records, which coincides with the increase in the differences between the predicted (dynamic fit) and optimized (time-specific fit) parameter values. The main differences occur for systemic arterial pressure. These are due to the predicted value of ${PaCO}_{2,n}$ being higher than the optimized value (Table 6, Pred. and Fitted values in record 6, respectively). The predicted value for this parameter is similar to the optimized value for the previous record, and its effect on many of the cardiovascular controller mechanisms is related to the inflection behavior of systemic arterial pressure at mid-stimulus levels (Equations of the Afferent Pathways, Blood Flow Local Control, and CNS Ischemic Response in the Supplementary Materials). Other significant differences are related to the lower slopes and the offset of BF and TI and could be related to a higher value of n and $P_{max}$ [45].

**Figure 7:**
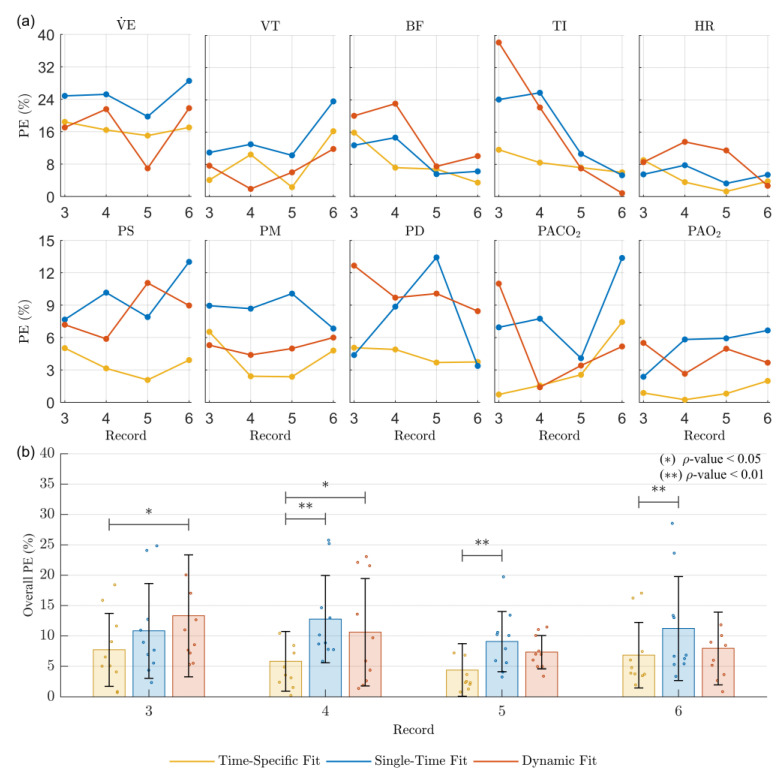
Percentage prediction error (PE) obtained for the physiological model responses regarding the DB2 records from third to sixth following three approaches: time-specific fit, single-time fit, and proposed dynamic fit. (**a**) presents the median of PE results of each approach, and (**b**) presents the overall PE results. The height of the bars corresponds to the overall PE, and the error bars indicate the standard deviation range. Statistically significant differences between the PE values of each approach were found using the Wilcoxon signed-rank test and are shown as (*) for ρ < 0.05 and (**) for ρ < 0.01.

Figure 7 shows the PE results obtained for the model predictions regarding the DB2 records from third to sixth. Figure 7a presents the median PE results of each approach for the variables evaluated. Figure 7b presents the overall PE results for each approach. The mean PE values for each evaluated variable are presented as individual points. The overall PE values and statistically significant differences between the results for each approach are also presented.

Table 7 presents the overall PE values for each fitting approach regarding the DB2 records from the third to the sixth. The values correspond to the heights reached by the bars plotted in Figure 7b.

The results for the third record corroborate the significant difference in the simulations related to the dynamic fit approach compared to those of the time-specific approach. The above evidence the high sensitivity of the dynamic fit approach regarding significant changes in the variables evaluated, similar to the time-specific fit results (Table 4). For this record, the significant changes mentioned evidence a limitation of the strategy proposed for DB2 regarding a personalized approach (Section 2.2.2. Experimental Data), considering the small number of samples used for the prediction (only two), which correspond to a different subject from the one for whom predictions are made.

The values presented for the fourth record evidence an improvement in the dynamic fit approach, even presenting better overall results than the traditional single-time fit approach. Although the distribution of the PE values is similar to that of the previous record, the improvement in the behavior of the variables allows results closer to those of the time-specific fit approach, mainly for the gas exchange variables.

The values for the dynamic fit approach in the fifth record also show better results than the traditional single-time fit approach, even for more variables than in the previous record. This consecutive improvement is related to the greater similarity regarding the magnitudes and behaviors of the variables (Figure 6), a consequence of the smaller overall difference between the predicted and optimized parameter values (Table 6).

The results for the sixth record show better results with the dynamic fit approach than the traditional single-time fit approach for most of the variables evaluated. The differences obtained regarding the time-specific fit approach are related to those described for the steady-state results and were expected considering the long prediction time for this record (Table 2).

The results in Table 7 differ from those in Table 4 for registers 3 to 6 in DB2 due to the input values used in each case. The input values in Table 4 are based on a distribution of $\dot{V}O_{2}$ and $\dot{V}{CO}_{2}$ that is specific to the AT value of each individual record. In contrast, in Table 7, the distribution of $\dot{V}O_{2}$ and $\dot{V}{CO}_{2}$ are defined from the AT value of the preceding record to maintain consistency with the dynamic fit approach. As a result, while the traditional single-time fit and the time-specific fit approaches both utilize identical input distribution values for an accurate comparison, the values diverge slightly due to the AT considered in each case.

## 4. Discussion

### 4.1. Time-Specific Model Fitting

It is highlighted that the highest deviations and errors are mainly related to the respiratory variables, contrary to what is presented for the gas exchange variables (Table 4). These results are similar to those reported in the building and validation of the case study model [28,33] and therefore could be mainly related to the predictability characteristics of the model rather than resulting from the method presented.

It was also evidenced that the variabilities and overall PE values were lower for DB2 (Table 4). These results are related to the characteristic dispersion of the DB1 records and show the better performance of the model under a personalized fitting perspective.

Regarding DB2, there were continuous decreases in the overall PE values between the first and second records and from the third to the fifth (Table 4). These results could be related to the sensitivity of the dynamic fitting strategy, which shows the records with significant changes in the cardiorespiratory response. Thus, considering the approximation proposed for DB2 (Section 2.2.2. Experimental Data), the model presents better results between successive records of the same subject due to the use and accumulation of previously optimized parameter values (which do not change regarding the next fit). Still, when there is a significant change in the system’s response, which in this case is due to the variation in the recorded subject, the prediction error of the variables increases. This is not observed for DB1, possibly due to the variability of the experimental data in its records.

### 4.2. Dynamic Parameter Modeling

It could be generally observed for DB2 that, as the number of records evaluated increased, the number of parameters that required re-modeling became increasingly smaller (Table 5 and Table 6). Even so, this trend is not fulfilled after evaluating the third record, a result that provides evidence for a significant alteration in the cardiorespiratory characteristics and which could be related to the change in the subject recorded in accordance with the approach implemented (Section 2.2.2. Experimental Data). In the case of DB1, despite the variability in a population record, this effect is attenuated considering the fit regarding the mean trend in the data.

It was also observed that the number of parameters that did not satisfy the physiological justification criteria was higher for DB2, and the highest number was presented for its sixth record (Table 5 and Table 6). The above also shows the sensitivity of the strategy to significant changes in the records, which for DB1 are attenuated considering the fit regarding the mean trend of the experimental data. For DB2, these results are related to greater difficulty modeling the temporal behavior of the parameters regarding the available factors and optimization records. This complexity increases, considering the longer prediction time for the sixth record (Table 2).

It should be noted that, as the number of records increases, the number of parameters to be modeled also increases. This is because the optimized parameters are not necessarily the same between consecutive model fits. According to the incorporated static fit strategy, the selection of the parameters involves an evaluation of the differences between the experimental data and the model predictions so that the variations between characteristics or magnitudes promote the optimization of the most related parameter.

Although a greater number of model parameters are consistent, the fact that all physiological characteristics change with time also implies a more significant computational load, which may not be justified regarding the temporal trends of the optimizations. ${\dot{P}}_{max}$ and ${VTCO}_{2}$, regarding DB2, are examples of the above, and although their values are modified in some of the records, they do not change again for subsequent ones, or their variations are not representative (Table 6).

### 4.3. Validation

Regarding the simulation comparisons for the third record of DB1, the higher overall PE value of the traditional single-time fit approach provides evidence of the change in the cardiorespiratory system over time and, therefore, the importance of a dynamic fit of the model to obtain more adequate predictions of the variables (Figure 5). Additionally, the greater similarity of the results related to the dynamic fit approach with those of the time-specific fit, both in magnitude and behavior (Figure 4), indicates the usefulness of considering the temporal trend of associated factors and parameter optimizations to predict the future state of the modeled system.

The dynamic fit approach results provided evidence of a greater sensitivity of the strategy regarding the personalized records (DB2) than with population records (DB1) (Figure 4, Figure 5, Figure 6 and Figure 7). This sensitivity is related to significant variation in the variables to be predicted regarding their previous records and the few samples used for the prediction. Although the dynamic fit approach considered the same number of samples regarding the third record of both databases, the variation in the temporal trend of the records of DB1 is less than DB2 due to the mean trend of the registered subjects. For DB2, the mentioned variation provides evidence for a limitation in the approximation used for the personalized records (Section 2.2.2. Experimental Data) since the records used for the prediction correspond to a different subject from the one attempted to predict.

As the number of temporary records evaluated for DB2 increases, the dynamic fit approach exhibits better overall results regarding accuracy and behavior than the traditional single-time fit approach (Figure 6 and Figure 7, Table 7). This performance can be mainly attributed to two characteristics of the proposed strategy. Firstly, using a more significant number of samples allows us to obtain model parameters with more precise predictions regarding the optimized results (Table 6). Secondly, using previously fitted parameters, some of which do not change between optimizations, allows us to obtain predictions with behaviors and magnitudes more similar to those of the time-specific fit to each record. The above confirms the advantage of dynamic fit regarding the traditional application of a fitted model in a single instant, in addition to evidence that a personalized approach is more appropriate than a population one.

Regarding the last DB2 record, despite the good overall result of the dynamic fit approach, the difference in the behavior of some variables regarding the time-specific fit approach simulations was highlighted (Figure 6). Mainly, this difference happened because using previous values of optimized parameters implies results with the characteristics of previous fittings (Table 6). In this sense, when evaluating a longer prediction time and, therefore, significant variations of the variables (due to the expected change of the modeled system to age), evidence could be provided of the memory characteristic of the proposed strategy.

### 4.4. Application, Limitations, and Future Work

In this work, a dynamic fitting strategy for physiological models was developed and evaluated by utilizing a model of the cardiorespiratory system as a case study. Experimental datasets from a longitudinal study of healthy subjects under aerobic exercise were used. The validation of the strategy consisted of comparing the model predictions using the proposed strategy against those obtained from the traditional approach, i.e., a population fit at a single instant of time. The main objective was to demonstrate the importance of dynamic fitting of the physiological model.

We conclude that our strategy is more effectively helpful in analyzing the cardiorespiratory system response than the traditional approach. This effectiveness is attributed to the consistently superior results regarding the predicted variables’ accuracy and behavior. This successful performance validates our strategy for both the model fitting at each point of time and predictions of future states of the physiological system. Furthermore, this evidence emphasizes the importance of considering the temporal evolution of physiological systems and contributing factors, such as lifestyle habits, accidents, illnesses, and clinical history. These elements play a significant role in the overall system, a fact which our strategy effectively integrates.

Our results underscore the practical utility of our proposed strategy in a clinical context, specifically in predictive, preventive, and personalized medicine. Essentially, our strategy serves as a tool that adapts mathematical models for analyzing and simulating the time-dependent responses of the physiological systems.

The proposal to include parameter variation due to natural life situations of a subject, such as changes in habits, diseases, or treatments to which they are subjected, constitutes significant advancement for personalized medicine. This approach acknowledges the dynamics of the evolutionary process of an individual regarding time. It offers the significantly ability to personalize a physiological model, enabling simulations and predictions of a subject’s current and future response to different stimuli, pathologies, and conditions. Furthermore, this approach aids the identification of the characteristics and mechanisms most closely linked to observed behaviors and values.

The results presented in this study indicate that the proposed approach outperformed the traditional one with statistically significant differences following a population perspective. Moreover, the accuracy of the predictive capability of the proposal showed improvements, as experimental data at different time states were added to analysis from a personalized perspective. These results were observed when adjusting the model parameters based on DB1 and DB2, which included three and six records from different time instants, respectively. While exercise stimulus was taken into account in this study as a safe and non-invasive procedure for healthy people (spanning in DB2 several years to validate the model parameter’s trends), this approach is also highly applicable in a clinical setting, especially for patients who are continuously monitored, such as those in intensive care units (ICUs).

This work also identified aspects that may limit the application of the proposed strategy. The sensitivity of the results regarding significant changes of variables to be predicted between records is highlighted, both for the dynamic fitting of the physiological model and for predicting the system’s future state. This aspect can be related to large time differences between consecutive records and specifically to the predictive approach with a short number of samples. The quality of the experimental data records is another crucial factor that can significantly influence the results. A dataset containing errors or inconsistencies could lead to inaccurate predictions and model fitting. Finally, the availability of adequate factors related to the temporal response of the system can influence the predictive approach’s outcomes.

The proposed strategy may have future applications due to the good validation results. Considering that, in this study, all the procedures were generically described, its application regarding other physiological systems, stimuli, characteristics of subjects, and experimental recording conditions can also be evaluated. Works related to the temporal analysis and the prediction of subjects with different pathologies, clinical treatments, medication regimens, or physical conditioning could be considered for the case study model.

According to the above, the strategy can be considered, for instance, in the field of mechanical ventilation therapy, applied to analyze the temporal changes in patients’ respiratory patterns, optimize ventilator settings in real time based on individual patients’ responses, and predict the future state of the patient’s respiratory system. It could be crucial to understand how mechanic ventilation (MV) affects the patient over time, leading to personalized ventilator settings that could significantly enhance patient comfort and outcomes while reducing the risk of ventilation-induced lung injuries. By applying our strategy, clinicians could potentially improve MV therapy by leveraging personalized, predictive data, paving the way for advancements in patient care and treatment efficacy.

Even though the presented strategy focuses mainly on the fitting of complex physiological models, it can also be applied to simpler cases. In this context, it should be considered that the minimum errors that can be obtained with the strategy will not be less than the values inherent to the model. Furthermore, the following is usually the case: the greater the complexity with which the model describes its interactions and functions, the greater the precision and accuracy of its predictions [13,14].

Although the proposed methodology was adequate to validate the strategy, some aspects could be considered for future studies. These include the evaluation of the effect of time between records; the assessment of different strategies for modeling the time trend of parameters; the identification, analysis, and evaluation of various factors related to the temporal response of the system to the predictive approach; and the definition and evaluation of criteria that allow us to define which parameters justify being modeled based on the time trend of their optimizations.

## 5. Conclusions

This paper presents the description, application, and validation of a dynamic fitting strategy for physiological models in settings that include a large number of parameters and a limited amount of experimental data. The proposed strategy consists of different procedures that allow the dynamic fit of the model; the modeling of the temporal trend of their parameters; and the validation, considering its usefulness, to predict the future state of the modeled system. The results allow us to conclude that our proposed strategy enhances the applicability of comprehensive mathematical models of physiological systems in a predictive, preventive, and personalized manner.

## Figures and Tables

**Table 1:** CR model symbol descriptions. This table lists and defines the abbreviations used in the CR model, complementing the schematic representation illustrated in Figure 1.

Abbreviation	Definition
AT	Anaerobic threshold
BF	Breathing frequency
$Cv{CO}_{2}$	Carbon dioxide concentration in the venous blood
$CvO_{2}$	Oxygen concentration in the venous blood
$E_{max,jv}$	End-systolic elastance of each ventricle
${FiCO}_{2}$	Inspired fractions of dry carbon dioxide
${FiO}_{2}$	Inspired fractions of dry oxygen
HR	Heart rate
I	Exercise intensity (central command action)
MRV	Metabolically related neural drive component of the ventilation
Nd	Neural drive signal
Pao	The pressure at the airway opening
$P_{atm}$	Atmospheric pressure
$Pb{CO}_{2}$	Brain carbon dioxide partial pressure
$P_{sa}$	Systemic arterial pressure
$R_{jp}$	Resistance related to each peripheral circulation
$T_{C}$	Total duration of the muscular contraction
$T_{im}$	Duration of the muscular contraction–relaxation cycle
$\dot{V}$	Airflow
$\dot{V}E$	Total minute ventilation
$\dot{V}{CO}_{2}$	Carbon dioxide output
$\dot{V}O_{2}$	Oxygen uptake
$V_{u,jv}$	Unstressed volume related to each venous circulation
VT	Tidal volume
TI	Inspiratory time
Psa	Systemic arterial pressure
$Q_{jp}$	Blood flow related to each peripheral circulation
$Q_{bp}$	Blood flow related to the brain circulation
$Q_{pp}$	Blood flow related to the pulmonary peripheral circulation
$PaCO_{2}$	Arterial carbon dioxide partial pressure
$PaO_{2}$	Arterial oxygen partial pressure

**Table 2:** Recorded information for each database of the experimental study. Data are reported as mean and standard deviation for the DB1.

Registered Information	DB1 Records			DB2 Records					
	1	2	3	1	2	3	4	5	6
Environmental conditions									
Ambient temperature (°C)	26.6 ± 1.5	27.0 ± 1.6	25.9 ± 1.3	25.0	26.0	28.0	25.0	27.0	26.0
Relative humidity (%)	63.7 ± 5.0	58.7 ± 4.5	61.8 ± 7.1	56.0	56.0	64.0	49.0	70.0	49.0
Subject data									
Age (years)	28.3 ± 6.8	29.0 ± 6.9	29.9 ± 6.9	25.2	26.0	29.9	30.8	31.5	63.1
Height (cm)	173.6 ± 6.3	173.6 ± 6.3	173.6 ± 6.3	163.0	163.0	167.4	167.4	167.4	165.0
Weight (kg)	76.7 ± 9.6	76.6 ± 10.8	77.6 ± 11.4	71.8	70.9	83.3	79.2	81.6	71.3
Body mass index (kg/$m^{2}$)	25.5 ± 2.8	25.4 ± 3.0	25.7 ± 3.2	27.0	26.7	29.7	28.3	29.1	26.2
Average sleep (hours per day)	6.6 ± 0.9	6.6 ± 1.0	6.7 ± 1.0	7.0	7.0	6.5	7.0	8.0	6.0
Average physical activity (hours per week)	6.4 ± 4.5	4.4 ± 4.5	3.3 ± 3.5	5.0	2.0	0.0	4.0	0.0	0.0

**Table 3:** Values of parameters calculated from experimental data.

Parameter	DB1			DB2					
	1	2	3	1	2	3	4	5	6
$V_{tot}$ (mL)	5025.9 ± 454.6 **	5022.2 ± 497.4 **	5062.4 ± 526.0 **	4642.0	4604.7	5185.1	5023.7	5118.7	4657.5
$AT\;for\;\dot{V}{CO}_{2}$ $\left(L/min\right)$	0.9 ± 0.5 *	0.7 ± 0.3 *	0.8 ± 0.6 *	0.9	0.8	1.0	1.2	1.4	1.4
${VT}_{n}$ (mL)	0.72 ± 0.12 **	0.76 ± 0.13 **	0.78 ± 0.13 **	0.60	0.73	0.77	0.67	0.66	0.74

* Values reported as mean and standard deviation. ** Values reported as median and interquartile distance.

**Table 4:** Prediction error (%) of the model fitted to each record (time-specific approach). Overall PE values are reported as median values, while the remaining variables are presented in median and interquartile range.

PE	DB1			DB2					
	1	2	3	1	2	3	4	5	6
Overall	7.57	8.42	8.03	8.51	6.13	7.24	5.25	5.13	6.84
$\dot{V}E$	7.40 ± 7.90	7.37 ± 9.81	7.19 ± 7.47	10.27 ± 2.63	15.07 ± 5.47	17.07 ± 9.93	14.89 ± 7.15	16.21 ± 2.73	17.05 ± 5.94
VT	11.10 ± 12.26	11.81 ± 15.53	12.56 ± 13.56	12.96 ± 16.26	1.76 ± 5.86	5.52 ± 9.69	12.99 ± 3.79	4.56 ± 11.39	16.23 ± 8.75
BF	10.08 ± 13.58	12.06 ± 13.12	9.83 ± 12.46	16.19 ± 14.23	10.31 ± 6.47	13.52 ± 14.61	4.64 ± 6.27	6.68 ± 5.97	3.49 ± 4.44
TI	10.41 ± 12.46	11.88 ± 13.57	10.03 ± 13.36	8.56 ± 10.17	7.83 ± 7.02	10.67 ± 11.78	3.88 ± 9.62	6.42 ± 5.06	6.03 ± 2.72
HR	10.50 ± 11.19	13.44 ± 14.18	12.81 ± 14.81	8.69 ± 5.84	11.05 ± 4.42	6.77 ± 13.87	2.26 ± 5.71	1.73 ± 2.85	3.79 ± 3.71
PS	5.08 ± 7.35	5.36 ± 6.04	6.41 ± 7.57	9.67 ± 9.54	2.45 ± 1.79	4.22 ± 4.25	2.70 ± 6.86	2.11 ± 2.88	3.91 ± 6.81
PM	5.47 ± 7.23	5.15 ± 7.10	7.00 ± 7.89	5.62 ± 4.87	2.31 ± 1.17	6.18 ± 3.84	3.46 ± 3.13	3.89 ± 8.28	4.80 ± 2.22
PD	8.94 ± 12.64	10.39 ± 10.05	7.49 ± 11.29	10.45 ± 10.12	8.54 ± 1.06	6.48 ± 5.31	5.23 ± 6.21	6.12 ± 10.60	3.74 ± 1.89
$PA{CO}_{2}$	4.29 ± 4.83	4.43 ± 4.37	4.38 ± 6.67	1.06 ± 1.43	1.80 ± 0.66	0.90 ± 0.96	2.04 ± 1.18	2.63 ± 1.22	7.44 ± 4.81
$PAO_{2}$	2.48 ± 3.10	2.33 ± 3.05	2.62 ± 3.34	1.65 ± 1.06	0.17 ± 0.13	1.09 ± 1.73	0.38 ± 1.31	0.98 ± 0.78	1.98 ± 1.28

**Table 5:** Parameter optimization trend and prediction results for DB1 records.

Parameter	DB1			
	1	2	3	
	Fitted	Fitted	Fitted	Pred.
Cardiovascular Controller				
$G_{T,v}$	0.0792	0.0886	0.0840	0.0967
$T_{0}$	0.6704	0.6712	0.6712	0.6709
$I_{0,met}$	0.4266	0.4266	0.3845	*
$I_{0,sp}$	0.650	0.461	0.461	0.550
${PaCO}_{2,n}$	41.5	41.5	39.0	*
$k_{met}$	0.180	0.173	0.162	0.172
Cardiovascular System				
${KR}_{lv}$	0.000437	0.000448	0.000475	0.000463
$L_{pa}$	0.000180	0.000181	0.000175	0.000176
$L_{sa}$	0.000220	0.000184	0.000236	0.000256
$R_{sa}$	0.0478	0.0488	0.0476	0.0479
Gas Exchange System				
$\alpha_{2}$	0.0559	0.0559	0.0609	*
$C_{1}$	8.80	9.21	8.84	9.02
$K_{2}$	194.40	207.10	207.10	196.29
${MRBCO}_{2}$	0.00074	0.00074	0.00106	0.00092
Respiratory Controller				
Kbg	17.42	17.69	18.97	17.99
${KcCO}_{2}$	0.2792	0.2967	0.2989	0.2923
${KpCO}_{2}$	0.20250	0.20238	0.20238	0.20241
${KpO}_{2}$	6.79 × 10^−9^	8.69 × 10^−9^	7.80 × 10^−9^	1.14 × 10^−8^
$\lambda_{1}$	0.876	0.825	0.819	0.838
$\lambda_{2}$	0.368	0.343	0.397	0.334
n	1.722	1.878	1.676	1.730
$P_{max}$	94.9	82.1	82.1	93.0
Respiratory Mechanics				
$E_{rs}$	27.47	24.37	25.56	28.08
$R_{rs}$	3.53	2.97	3.08	3.15

* Parameters that were not predicted for the record.

**Table 6:** Parameter optimization trend and prediction results for DB2 records.

Parameter	DB2									
	1	2	3		4		5		6	
	Fitted	Fitted	Fitted	Pred.	Fitted	Pred.	Fitted	Pred.	Fitted	Pred.
Cardiovascular Controller										
$G_{T,s}$	−0.130	−0.130	−0.130	−0.130	−0.092	*	−0.092	**	−0.092	−0.113
$G_{T,v}$	0.102	0.108	0.081	0.109	0.081	0.066	0.045	0.041	0.054	**
$G_{R_{ep}}$	1.940	1.940	1.940	1.940	1.876	*	1.876	2.432	1.613	**
$T_{0}$	0.588	0.588	0.549	*	0.581	0.542	0.571	0.631	0.571	**
$I_{0,met}$	0.55254	0.55254	0.55360	*	0.55360	0.55384	0.55360	0.55503	0.55360	0.55360
$k_{met}$	0.180	0.182	0.182	0.189	0.182	0.181	0.182	0.178	0.182	0.182
${PaCO}_{2,n}$	40.0	40.0	46.2	*	46.2	**	55.4	51.8	39.8	58.3
$P_{n}$	117.3	96.2	74.5	**	92.6	**	92.6	68.4	92.3	90.7
$\Phi_{max}$	22.03	22.03	26.00	*	26.00	26.47	26.00	25.25	26.00	26.00
Cardiovascular System										
${KE}_{lv}$	0.0105	0.0098	0.0098	0.0068	0.0098	0.0099	0.0098	0.0109	0.0098	0.0097
${KE}_{rv}$	0.0116	0.0078	0.0078	**	0.0079	0.0078	0.0087	0.0071	0.0109	**
${KR}_{lv}$	0.000477	0.000469	0.000486	0.000434	0.000363	0.000488	0.000488	**	0.000431	0.000233
${KR}_{rv}$	0.0014	0.0014	0.0014	0.0014	0.0013	*	0.0013	**	0.0013	**
$R_{sa}$	0.053	0.054	0.053	0.055	0.053	0.053	0.046	0.052	0.090	**
Gas Exchange System										
$a_{2}$	1.819	1.819	1.819	1.819	1.819	1.819	1.819	1.819	2.365	*
$\alpha_{2}$	0.05591	0.05591	0.05591	0.05591	0.06029	*	0.06029	**	0.06029	0.05792
$C_{1}$	11.10	11.10	10.65	10.68	10.14	10.54	9.64	**	10.05	9.64
$C_{2}$	87.00	93.77	93.77	88.29	93.77	93.76	93.77	**	93.77	**
$K_{2}$	194.40	194.40	149.91	*	149.91	**	149.91	149.91	149.91	**
${MRBCO}_{2}$	0.00098	0.00081	0.00075	**	0.00063	0.00076	0.00066	0.00053	0.00065	**
${VTCO}_{2}$	15.0	15.0	15.0	15.0	15.0	15.0	15.1	*	15.1	**
Respiratory Controller										
Kbg	25.13	22.98	22.73	24.65	20.72	21.33	21.76	21.95	20.33	**
${KcCO}_{2}$	0.4385	0.4573	0.4481	0.5398	0.4559	0.4167	0.4664	0.4593	0.4664	0.5005
${KpCO}_{2}$	0.20250	0.17739	0.16777	**	0.16777	0.16555	0.15523	**	0.19200	**
${KpO}_{2}$	2.69 × 10^−9^	3.76 × 10^−9^	3.85 × 10^−9^	**	2.36 × 10^−9^	**	4.80 × 10^−9^	3.06 × 10^−9^	3.54 × 10^−9^	**
$\lambda_{1}$	0.458	0.752	0.899	**	0.609	0.933	0.609	**	1.099	**
$\lambda_{2}$	0.468	0.245	0.691	**	0.348	0.794	0.348	**	0.348	**
n	1.362	0.834	0.899	**	1.123	1.794	1.016	**	1.348	**
${\dot{P}}_{max}$	1055.4	1055.4	1069.8	*	1069.8	1072.9	1069.8	1069.8	1069.8	1069.8
$P_{max}$	72.2	47.7	51.2	68.5	63.5	90.1	93.9	**	58.7	**
${V0}_{dead}$	0.2381	0.2278	0.2381	0.1831	0.2273	0.2358	0.2273	0.3339	0.2319	0.2057
Respiratory Mechanics										
$E_{rs}$	19.86	29.00	29.00	21.60	26.72	27.14	29.00	29.01	26.21	**
$R_{rs}$	3.06	3.06	3.50	*	3.50	3.60	3.50	4.10	3.50	3.50

* Parameters that were not predicted for the record. ** Predicted values did not satisfy the physiological justification criteria.

**Table 7:** Overall PE values for DB2 records from third to sixth. The values are presented as mean and standard deviation.

Fitting Approach	Overall PE Values (%) for Records			
	3	4	5	6
Time-Specific Fit	7.73 ± 5.99	5.83 ± 4.90	4.42 ± 4.31	6.84 ± 5.37
Single-Time Fit	10.84 ± 7.79	12.76 ± 7.18	9.08 ± 4.93	11.24 ± 8.54
Dynamic Fit	13.32 ± 10.02	10.62 ± 8.83	7.34 ± 2.73	7.95 ± 5.97

## Data Availability

The complete description of the procedures and characteristics of the experimental design is reported in the paper on the model building and evaluation [33]. The information required to replicate the cardiorespiratory model and the static fitting strategy can be found in the previous papers [28,33]. The equations and parameter values to implement and replicate the proposed strategy are described throughout this work.

## Supplementary Materials

The following supporting information can be downloaded at: https://www.mdpi.com/article/10.3390/jpm14040406/s1. Model equations: S1. Cardiovascular System, S2. Cardiovascular Controller, S3. Respiratory mechanics, S4. Respiratory controller, S5. Gas exchange system; Table S1: Parameters of Systemic Circulation; Table S2: Parameters of Pulmonary Circulation; Table S3: Parameters of the Heart; Table S4: Parameters of Afferent Pathways - Afferent Baroreflex Pathway; Table S5: Parameters of Afferent Pathways-Afferent Chemoreflex Pathway; Table S6: Parameters of Blood Flow Local Control: Cerebral Blood Flow; Table S7: Parameters of Blood Flow Local Control-Coronary and Resting Muscle Blood Flow; Table S8: Parameters of Blood Flow Local Control: Active Muscle Blood Flow; Table S9: Parameters of CNS Ischemic Response; Table S10: Parameters of Efferent Pathways; Table S11: Parameters of Effectors for Reflex Control-Resistances, Unstressed Volumes and Cardiac Elastances; Table S12: Parameters of Effectors for Reflex Control-Heart Period; Table S13: Parameters of Ventilation Controller; Table S14: Parameters of Breathing Pattern Optimizer; Table S15: Parameters of the Gas Exchange and Mixing; Table S16: Parameters of the Gas transport: Brain Compartment; Table S17: Parameters of the Gas transport-Body Tissues Compartment; Table S18: Parameters of the Gas transport-Metabolism Dynamic. Reference [33] is cited in Supplementary Materials.
