# Supplementary material for: A Dynamic Fitting Strategy for Physiological Models: A Case Study of a Cardiorespiratory Model for the Simulation of Incremental Aerobic Exercise
Source: J Pers Med. 2024 Apr 11;14(4):406. doi: 10.3390/jpm14040406 (PMC11051577; doi:10.3390/jpm14040406)
Supplement: Supplementary file 1 [file jpm-14-00406-s001.zip › jpm-2914998-supplementary/SupplemetaryMaterial.pdf]

TITLE

Supplementary Material for “A dynamic fitting strategy for physiological models: A case study of a cardiorespiratory model for the simulation of incremental aerobic exercise”.

AUTHORS

Names

Carlos A. Sarmiento*, Alher M. Hernández, Miguel Á. Mañanas, Leidy Y. Serna

Correspondence Information

- Corresponding author: Carlos Andrés Sarmiento
- Address: Bioinstrumentation and Clinical Engineering Research Group, Bioengineering Department, Engineering Faculty,
- Universidad de Antioquia UdeA, Calle 70 # 52-51, Medellin 050016, Colombia.
- E-mail address: carlos.sarmiento@dea.edu.co

MODEL EQUATIONS

The equations of the cardiorespiratory model used as a case study and related with the manuscript are presented bellow. Full details of each of the systems and controllers' equations, and the nominal values of the parameters can be consulted in the previous work [1].

S1. Cardiovascular System

The equations at different circulatory system points are presented below. The main elements include compliances (C), inertances (L), pressures (P), blood flows (Q), hydraulic resistances (R) and blood volumes (V). Multiple subscripts are used to indicate a specific compartment: sa, systemic arteries; vc, vena cava; pa, pulmonary arteries; pp, pulmonary peripheral circulation; pv, pulmonary veins; la, left atrium; lv, left ventricle; ra, right atrium; rv, right ventricle. The systemic circulation describes peripheral and venous circulation (subscripts p and v) in six compartments: extra-splanchnic (e), splanchnic (s), brain (b), coronary (c), skeletal resting muscle (rm) and active skeletal muscle (am). $\mathrm{P_{abd}}$, $\mathrm{P_{im}}$ and $\mathrm{P_{thor}}$ are the abdominal pressure, the extravascular pressure of the active muscle veins (i.e., the intramuscular pressure) and the intrathoracic pressure, respectively. The subscripts i, n, max, min, T, and u are used to indicate input, nominal, maximum, minimum, total, and unstressed values, respectively.

S1.1. Systemic Circulation

$$L_{sa} \cdot \frac{\mathrm{dQ_{sa}}}{\mathrm{dt}} = (\mathrm{P_{sa}} - \mathrm{P_{thor}}) - R_{sa} \cdot \mathrm{Q_{sa}} - \mathrm{P_{sp}} \qquad \text{(S1)}$$

Table S1. Parameters of Systemic Circulation.

Parameter	Definition
R_{sa}	Systemic arterial hydraulic resistance
L_{sa}	Systemic arterial inertance

S1.2. Pulmonary Circulation

$$L_{pa} \cdot \frac{\mathrm{dQ_{pa}}}{\mathrm{dt}} = \mathrm{P_{pa}} - R_{pa} \cdot \mathrm{Q_{pa}} - \mathrm{P_{pp}} \qquad \text{(S2)}$$

Table S2. Parameters of Pulmonary Circulation.

Parameter	Definition
L_{pa}	Pulmonary arterial inertance
R_{pa}	Pulmonary arterial flow resistance

S1.3. The Heart

$$\mathrm{R_{lv}} = KR_{lv} \cdot \mathrm{P_{max,lv}} \qquad \text{(S3)}$$

$$\mathrm{P_{max,lv}(t)} = \varphi(\mathrm{t}) \cdot \mathrm{E_{max,lv}} \cdot \left(\mathrm{V_{lv}} - V_{u,lv}\right) + [1 - \varphi(\mathrm{t})] \cdot P_{0,lv} \cdot \left(\exp^{(K_{E,lv} \cdot \mathrm{V_{lv}})} - 1\right) \quad 0 \le \varphi(\mathrm{t}) \le 1 \qquad \text{(S4)}$$

$$\mathrm{R_{rv}} = KR_{rv} \cdot \mathrm{P_{max,rv}} \qquad \text{(S5)}$$

$$\mathrm{P_{max,rv}(t)} = \varphi(\mathrm{t}) \cdot \mathrm{E_{max,rv}} \cdot \left(\mathrm{V_{rv}} - V_{u,rv}\right) + [1 - \varphi(\mathrm{t})] \cdot P_{0,rv} \cdot \left(\exp^{(K_{E,rv} \cdot \mathrm{V_{rv}})} - 1\right) \quad 0 \le \varphi(\mathrm{t}) \le 1 \qquad \text{(S6)}$$

Where $\mathrm{W_{h,lv}}$ and $\mathrm{W_{h,rv}}$ are the average power of the cardiac pump for the left and right ventricles; $\mathrm{E_{max,lv}}$ and $\mathrm{E_{max,rv}}$ are the left and right ventricular elastance at the instant of maximum contraction; $\varphi(\mathrm{t})$ is the ventricle activation function, with $\varphi(\mathrm{t}) = 1$ at maximum contraction and $\varphi(\mathrm{t}) = 0$ at complete relaxation.

Table S3. Parameters of the Heart.

Parameter	Definition
$K_{E,lv}$	Parameters that describe the end-diastolic pressure-volume relationship in the left ventricle
$K_{E,rv}$	Parameters that describe the end-diastolic pressure-volume relationship in the Right ventricle
KR_{lv}	Parameters that mimic the viscosity of the left ventricle
KR_{rv}	Parameters that mimic the viscosity of the right ventricle
$P_{0,lv}$	Parameters that describe the end-diastolic pressure-volume relationship in the left ventricle
$P_{0,rv}$	Parameters that describe the end-diastolic pressure-volume relationship in the right ventricle
$V_{u,lv}$	Left ventricular unstressed volume
$V_{u,rv}$	Right ventricular unstressed volume

S2. Cardiovascular Controller

The equations describing the mechanisms of cardiovascular controller are presented below.

S2.1. Afferent Pathways

Afferent Baroreflex Pathway

$$\tau_P \cdot \frac{\mathrm{d}\widetilde{\mathrm{P}}}{\mathrm{dt}} = \mathrm{P_{sa}} + \tau_Z \cdot \frac{\mathrm{dP_{sa}}}{\mathrm{dt}} - \widetilde{\mathrm{P}} \qquad \text{(S7)}$$

$$\mathrm{f_{ab}} = \frac{f_{ab,min} + f_{ab,max} \cdot \exp^{\left(\frac{\tilde{\mathrm{P}} - P_n}{k_{ab}}\right)}}{1 + \exp^{\left(\frac{\tilde{\mathrm{P}} - P_n}{k_{ab}}\right)}} \tag{S8}$$

Where $\mathrm{f_{ab}}$ is the afferent activity from baroreceptors.

Table S4. Parameters of Afferent Pathways: Afferent Baroreflex Pathway.

Parameter	Definition
$f_{ab,max}$	Upper saturation level of the frequency discharge in the baroreceptor afferent fibers
$f_{ab,min}$	Lower saturation level of the frequency discharge in the baroreceptor afferent fibers
k_{ab}	Parameter related to the slope of the static function at the central point
P_n	Value of baroreceptor pressure at the central point of the sigmoidal function
τ_P	Time constant for the real pole
τ_Z	Time constant for the real zero

Afferent Chemoreflex Pathway

$$\varphi_{\mathrm{ac}} = \frac{f_{ac,max} + f_{ac,min} \cdot \exp^{\left(\frac{\mathrm{PaO_2} - PaO_{2ac,n}}{k_{ac}}\right)}}{1 + \exp^{\left(\frac{\mathrm{PaO_2} - PaO_{2ac,n}}{k_{ac}}\right)}} \cdot \left[\mathrm{K} \cdot \ln\left(\frac{\mathrm{PaCO_2}}{PaCO_{2,n}}\right) + f_{acCO_2,n}\right] \tag{S9}$$

$$\mathrm{K} = \begin{cases} K_H & \mathrm{PaO_2} > 80 \\ K_H - 1.2 \cdot \left(\frac{\mathrm{PaO_2} - 80}{30}\right) & 40 < \mathrm{PaO_2} < 80 \\ K_H - 1.6 & \mathrm{PaO_2} < 40 \end{cases} \tag{S10}$$

$$\tau_{ac} \cdot \frac{\mathrm{df_{ac}}}{\mathrm{dt}} = \varphi_{\mathrm{ac}} - \mathrm{f_{ac}} \tag{S11}$$

Where $\mathrm{f_{ac}}$ is the afferent activity from chemoreceptors.

Table S5. Parameters of Afferent Pathways: Afferent Chemoreflex Pathway.

Parameter	Definition
$f_{acCO_2,n}$	Constant parameter tuned to reproduce the CO_2 static response
$f_{ac,max}$	Upper saturation level of the frequency discharge in the chemoreceptor afferent fibers
$f_{ac,min}$	Lower saturation level of the frequency discharge in the chemoreceptor afferent fibers
k_{ac}	Parameter related to the slope of the sigmoid at the central point
K_H	Constant parameter tuned to reproduce the CO_2 static response
$PaO_{2ac,n}$	Arterial PO_2 at the central point of the sigmoid
$PaCO_{2,n}$	$PaCO_2$ basal value
τ_{ac}	Time constant of the chemoreceptor mechanism

S2.2. Blood Flow Local Control

Cerebral Blood Flow

$$\Phi_{\mathrm{b}} = \frac{A + \frac{B}{1 + C \cdot \exp^{(D \cdot \log(\mathrm{PaCO_2}))}}}{A + \frac{B}{1 + C \cdot \exp^{(D \cdot \log(PaCO_2 n))}}} - 1 \tag{S12}$$

$$\tau_{CO_2} \cdot \frac{\mathrm{dx_{b,CO_2}}}{\mathrm{dt}} = -\mathrm{x_{b,CO_2}} - \Phi_{\mathrm{b}} \tag{S13}$$

Where $PaCO_2$ is the arterial carbon dioxide partial pressure and $\mathrm{x_{b,CO_2}}$ is a state variable representing the effect of CO_2 on cerebral circulation.

Table S6. Parameters of Blood Flow Local Control: Cerebral Blood Flow.

Parameter	Definition
A	Constant parameter
B	Constant parameter
C	Constant parameter
D	Constant parameter
τ_{CO_2}	Time constants of the effect of CO_2 on cerebral circulation

Coronary and Resting Muscle Blood Flow

$$\mathrm{R_{jp}} = R_{jpn} \cdot \frac{(1 + \mathrm{x_{j,CO_2}})}{(1 + \mathrm{x_{j,O_2}})} \tag{S14}$$

$$\tau_{O_2} \cdot \frac{dx_{j,O_2}}{dt} = -x_{j,O_2} - g_{j,O_2} \cdot \left(C_{vj,O_2} - C_{vj,O_2n}\right) \quad (S15)$$

$$C_{vj,O_2} = C_{aO_2} - \frac{M_{O_2,jp}}{Q_{jp}} \quad (S16)$$

$$\Phi_j = \frac{1 - \exp^{\left(\frac{PaCO_2 - PaCO_2n}{K_{j,CO2}}\right)}}{1 + \exp^{\left(\frac{PaCO_2 - PaCO_2n}{K_{j,CO2}}\right)}} \quad (S17)$$

$$\tau_{CO_2} \cdot \frac{dx_{j,CO_2}}{dt} = -x_{j,CO_2} + \Phi_j \quad (S18)$$

Where the subscript j indicates the systemic compartment, h for coronary and rm for skeletal resting muscle; x_{j,O_2} and x_{j,CO_2} are state variables representing the effect of O_2 and CO_2 on coronary and resting muscular circulation.

Table S7. Parameters of Blood Flow Local Control: Coronary and Resting Muscle Blood Flow.

Parameter	Definition
C_{vh,O_2n}	O_2 concentration in venous blood leaving the heart under normal conditions
C_{vrm,O_2n}	O_2 concentration in venous blood leaving the skeletal resting muscle under normal conditions
g_{h,O_2}	Constant gain factor
g_{rm,O_2}	Constant gain factor
$K_{h,CO2}$	Parameter related to the slope of the sigmoidal function at the central point
$K_{rm,CO2}$	Parameter related to the slope of the sigmoidal function at the central point
$M_{O_2,hpn}$	Nominal value of O_2 consumption rate in the heart
$M_{O_2,rmp}$	Consumption rate in the resting muscle
R_{hpn}	Normal peripheral resistance in coronary compartment

Active Muscle Blood Flow

$$\Phi_{met}(t) = \frac{\Phi_{min} + \Phi_{max} \cdot \exp^{\left(\frac{I - I_{0,met}}{k_{met}}\right)}}{1 + \exp^{\left(\frac{I - I_{0,met}}{k_{met}}\right)}} \quad (S19)$$

Where I is the action of central command and Φ_{met} is the output of the static sigmoidal characteristic.

Table S8. Parameters of Blood Flow Local Control: Active Muscle Blood Flow.

Parameter	Definition
$I_{0,met}$	Is I at the central point of the sigmoid
k_{met}	Parameter related to the slope of the sigmoid at the central point
Φ_{max}	Upper saturation of the static sigmoidal characteristic
Φ_{min}	Lower saturation of the static sigmoidal characteristic

S2.3. CNS Ischemic Response

$$w_{sj} = \frac{x_{sj}}{1 + \exp^{\left(\frac{PaO_2 - \tilde{P}O_{2,sj}}{k_{isc,sj}}\right)}} \quad (S20)$$

$$\tau_{isc} \cdot \frac{d\Delta\theta O_{2,sj}}{dt} = -\Delta\theta O_{2,sj} + w_{sj} \quad (S21)$$

$$\tau_{cc} \cdot \frac{d\Delta\theta CO_{2,sj}}{dt} = -\Delta\theta CO_{2,sj} + g_{ccsj} \cdot (PaCO_2 - PaCO_{2n}) \quad (S22)$$

$$\theta_{sj} = \theta_{sjn} - \Delta\theta O_{2,sj} - \Delta\theta CO_{2,sj} \quad (S23)$$

Where the subscript j=p, v, h indicates the response for peripheral resistances, veins, and heart, respectively; x_{sj} is the saturation of the hypoxic response; $\Delta\theta O_{2,sj}$ represents the change in the offset term caused by the CNS hypoxia; $\Delta\theta CO_{2,sj}$ represents the change in the offset term caused by central chemoreceptor stimulation.

Table S9. Parameters of CNS Ischemic Response.

Parameter	Definition
g_{ccsh}	Constant gain factor tuned to reproduce experimental results
g_{ccsp}	Constant gain factor tuned to reproduce experimental results
g_{ccsv}	Constant gain factor tuned to reproduce experimental results
$k_{isc,sh}$	Parameter related to the slope of the static function at the central point for heart
$k_{isc,sp}$	Parameter related to the slope of the static function at the central point for peripheral resistance
$k_{isc,sv}$	Parameter related to the slope of the static function at the central point for unstressed volume of veins
$\tilde{P}O_{2,sh}$	Value of PO_2 at the central point of the sigmoidal function for heart
$\tilde{P}O_{2,sp}$	Value of PO_2 at the central point of the sigmoidal function for peripheral resistance
$\tilde{P}O_{2,sv}$	Value of PO_2 at the central point of the sigmoidal function for unstressed volume of veins

Parameter	Definition
τ_{cc}	Time constant
τ_{isc}	Time constant of the mechanism
θ_{shn}	Offset term in basal condition for heart
θ_{spn}	Offset term in basal condition for peripheral resistance
θ_{svn}	Offset term in basal condition for unstressed volume of veins
x_{sh}	Saturation of the hypoxic response for heart
x_{sp}	Saturation of the hypoxic response for peripheral resistance
x_{sv}	Saturation of the hypoxic response for unstressed volume of veins

S2.4. Efferent Pathways

$$\mathrm{f_{sj}} = \begin{cases} f_{es,\infty} + \left(f_{es,0} - f_{es,\infty}\right) \cdot \exp^{\left(k_{es} \cdot \mathrm{f_{asj}}\right)} + \gamma_{\mathrm{sj}}(\mathrm{I}) & \mathrm{f_{sj}} < f_{es,max} \\ f_{es,max} & \mathrm{f_{sj}} \geq f_{es,max} \end{cases} \tag{S24}$$

$$\mathrm{f_{asj}} = W_{t,sj} \cdot \mathrm{Nt} + W_{b,sj} \cdot \mathrm{f_{ab}} + W_{c,sj} \cdot \mathrm{f_{ac}} + W_{p,sj} \cdot \mathrm{f_{ap}} - \theta_{\mathrm{sj}} \tag{S25}$$

$$\mathrm{f_v} = \frac{f_{ev,0} + f_{ev,\infty} \cdot \exp^{\left(\frac{\mathrm{f_{ab}} - f_{ab,0}}{k_{ev}}\right)}}{1 + \exp^{\left(\frac{\mathrm{f_{ab}} - f_{ab,0}}{k_{ev}}\right)}} - W_{t,v} \cdot \mathrm{Nt} + W_{c,v} \cdot \mathrm{f_{ac}} + W_{p,v} \cdot \mathrm{f_{ap}} - \theta_v + \gamma_{\mathrm{v}}(\mathrm{I}) \tag{S26}$$

$$\gamma_{\mathrm{i}} = \frac{\gamma_{i,min} + \gamma_{i,max} \cdot \exp^{\left(\frac{\mathrm{I} - I_{0,i}}{k_{cc,i}}\right)}}{1 + \exp^{\left(\frac{\mathrm{I} - I_{0,i}}{k_{cc,i}}\right)}} \tag{S27}$$

Where the subscript j=h, p, v indicates sympathetic activity to heart, peripheral resistances, and veins, respectively; the subscript i=sp, sv and sh indicate sympathetic activity to peripheral arteries, veins and heart, and v denotes vagal activity; f_{sj} is the sympathetic activity; f_v is the frequency of spike in the efferent vagal fibers.

Table S10. Parameters of Efferent Pathways.

Parameter	Definition
$f_{ab,0}$	Central value in the curve of f_{ab}
$f_{es,0}$	Constant parameter
$f_{es,\infty}$	Constant parameter
$f_{es,max}$	Upper saturation level above for the sympathetic activity
$f_{ev,0}$	Constant parameter
$f_{ev,\infty}$	Constant parameter
k_{es}	Constant parameter
k_{ev}	Constant parameter
$I_{0,sh}$	Value of exercise intensity at the central point of the sigmoid
$I_{0,sp}$	Value of exercise intensity at the central point of the sigmoid
$I_{0,sv}$	Value of exercise intensity at the central point of the sigmoid
$I_{0,v}$	Value of exercise intensity at the central point of the sigmoid
$k_{cc,sh}$	Parameter related to the slope at the central point
$k_{cc,sp}$	Parameter related to the slope at the central point
$k_{cc,sv}$	Parameter related to the slope at the central point
$k_{cc,v}$	Parameter related to the slope at the central point
$\gamma_{sh,max}$	Upper saturation of the central command response
$\gamma_{sh,min}$	Lower saturation of the central command response
$\gamma_{sp,max}$	Upper saturation of the central command response
$\gamma_{sp,min}$	Lower saturation of the central command response
$\gamma_{sv,max}$	Upper saturation of the central command response
$\gamma_{sv,min}$	Lower saturation of the central command response
$\gamma_{v,max}$	Upper saturation of the central command response
$\gamma_{v,min}$	Lower saturation of the central command response
θ_v	Offset term
$W_{b,sh}$	Synaptic weight tuned to reproduce physiological results
$W_{b,sp}$	Synaptic weight tuned to reproduce physiological results
$W_{b,sv}$	Synaptic weight tuned to reproduce physiological results
$W_{c,sh}$	Synaptic weight tuned to reproduce physiological results
$W_{c,sp}$	Synaptic weight tuned to reproduce physiological results
$W_{c,sv}$	Synaptic weight tuned to reproduce physiological results

Parameter	Definition
$W_{c,v}$	Synaptic weight tuned to reproduce physiological results
$W_{p,sh}$	Synaptic weight tuned to reproduce physiological results
$W_{p,sp}$	Synaptic weight tuned to reproduce physiological results
$W_{p,sv}$	Synaptic weight tuned to reproduce physiological results
$W_{p,v}$	Synaptic weight tuned to reproduce physiological results
$W_{t,sh}$	Synaptic weight tuned to reproduce physiological results
$W_{t,sp}$	Synaptic weight tuned to reproduce physiological results
$W_{t,sv}$	Synaptic weight tuned to reproduce physiological results
$W_{t,v}$	Synaptic weight tuned to reproduce physiological results

S2.5. Effectors for Reflex Control

Resistances, Unstressed Volumes and Cardiac Elastances

$$\sigma_\theta = \begin{cases} G_\theta \cdot \ln\left[f_{sj}(t - D_\theta) - f_{es,min} + 1\right] & f_{sj} \geq f_{es,min} \\ 0 & f_{sj} < f_{es,min} \end{cases} \quad \text{(S28)}$$

$$\tau_\theta \cdot \frac{d\Delta\theta}{dt} = -\Delta\theta + \sigma_\theta \quad \text{(S29)}$$

$$\theta(t) = \Delta\theta(t) + \theta_0 \quad \text{(S30)}$$

Where the subscript θ=R_{ep}, R_{sp}, $R_{rmp,n}$, $R_{amp,n}$, $V_{u,ev}$, $V_{u,sv}$, $V_{u,rmv}$, $V_{u,amv}$, $E_{max,lv}$, $E_{max,rv}$ denotes the generic controlled variable; σ_θ is the output of the static characteristic.

Table S11. Parameters of Effectors for Reflex Control: Resistances, Unstressed Volumes and Cardiac Elastances.

Parameter	Definition
$D_{E_{max,lv}}$	Pure latency of the mechanism
$D_{E_{max,rv}}$	Pure latency of the mechanism
$D_{R_{amp}}$	Pure latency of the mechanism
$D_{R_{ep}}$	Pure latency of the mechanism
$D_{R_{rmp}}$	Pure latency of the mechanism
$D_{R_{sp}}$	Pure latency of the mechanism
$D_{V_{amv}}$	Pure latency of the mechanism
$D_{V_{ev}}$	Pure latency of the mechanism
$D_{V_{rmv}}$	Pure latency of the mechanism
$D_{V_{sv}}$	Pure latency of the mechanism
$E_{max0,lv}$	Basal level of maximum end-systolic elastance of the left ventricle
$E_{max0,rv}$	Basal level of maximum end-systolic elastance of the right ventricle
$f_{es,min}$	Threshold for sympathetic stimulation
$G_{E_{max,lv}}$	Constant gain factor
$G_{E_{max,rv}}$	Constant gain factor
$G_{R_{amp}}$	Constant gain factor
$G_{R_{ep}}$	Constant gain factor
$G_{R_{rmp}}$	Constant gain factor
$G_{R_{sp}}$	Constant gain factor
$G_{V_{amv}}$	Constant gain factor
$G_{V_{ev}}$	Constant gain factor
$G_{V_{rmv}}$	Constant gain factor
$G_{V_{sv}}$	Constant gain factor
$R_{amp,0}$	Basal level of active skeletal peripheral resistance
$R_{ep,0}$	Basal level of extra-splanchnic peripheral resistance
$R_{rmp,0}$	Basal level of resting skeletal peripheral resistance
$R_{sp,0}$	Basal level of splanchnic peripheral resistance
$\tau_{E_{max,lv}}$	Time constant
$\tau_{E_{max,rv}}$	Time constant
$\tau_{R_{amp}}$	Time constant
$\tau_{R_{ep}}$	Time constant
$\tau_{R_{rmp}}$	Time constant
$\tau_{R_{sp}}$	Time constant
$\tau_{V_{amv}}$	Time constant
$\tau_{V_{ev}}$	Time constant
$\tau_{V_{rmv}}$	Time constant

Parameter	Definition
$\tau_{V_{sv}}$	Time constant
$V_{u,amv0}$	Basal level of active skeletal muscle venous unstressed volume
$V_{u,ev0}$	Basal level of extra-splanchnic venous unstressed volume
$V_{u,rmv0}$	Basal level of resting skeletal muscle venous unstressed volume
$V_{u,sv0}$	Basal level of splanchnic venous unstressed volume

Where v is spikes/s.

Heart Period

$$\sigma_{T,s}(t) = \begin{cases} G_{T,s} \cdot \ln\left[f_{sh}\left(t - D_{T,s}\right) - f_{es,min} + 1\right] & f_{sh} \geq f_{es,min} \\ 0 & f_{sh} < f_{es,min} \end{cases} \quad (S31)$$

$$\tau_{T,s} \cdot \frac{d\Delta T_s(t)}{dt} = -\Delta T_s(t) + \sigma_{T,s}(t) \quad (S32)$$

$$\sigma_{T,v}(t) = G_{T,v} \cdot f_v\left(t - D_{T,v}\right) \quad (S33)$$

$$\tau_{T,v} \cdot \frac{d\Delta T_v(t)}{dt} = -\Delta T_v(t) + \sigma_{T,v}(t) \quad (S34)$$

$$T = \Delta T_v + \Delta T_s + T_0 \quad (S35)$$

$$HR = \frac{1}{T} \quad (S36)$$

Where ΔT_v represents the changes in heart period induced by parasympathetic stimulation; ΔT_s represents the changes in heart period induced by sympathetic stimulation; $\sigma_{T,s}$ and $\sigma_{T,v}$ are the output of the static characteristic.

Table S12. Parameters of Effectors for Reflex Control: Heart Period.

Parameter	Definition
$D_{T,s}$	Pure latency of the mechanism
$D_{T,v}$	Pure latency of the mechanism
$f_{sh}IC$	Initial condition for the efferent sympathetic cardiac activity
f_vIC	Initial condition for the efferent vagal activity
$G_{T,s}$	Constant gain factor
$G_{T,v}$	Constant gain factor
T_0	Heart period in the absence of cardiac innervation
$\tau_{T,s}$	Time constant
$\tau_{T,v}$	Time constant

S3. Respiratory mechanics

The equations related to the Respiratory Mechanics are presented below. The main elements include compliances (C), elastances (E), pressures (P), resistances (R), volumes (V) and flows ($\dot{V}$). Different subscripts are used: AW, airway; CW, chest wall; L, lung transmural; LA, lower airway; pl, pleural; ua, upper airway.

S3.1. Pulmonary Mechanics

$$\dot{V}(t) = \frac{G_{AW}}{R_{rs}} \cdot \left((P_{musc}(t) - Pao) - E_{rs} \cdot V(t)\right) \quad (S37)$$

Where E_{rs} is the overall elastance; R_{rs} is the overall resistance; $P_{musc}(t)$ is the muscle pressure signal; Pao is the pressure at the airway opening; $\dot{V}(t)$ is the respiratory airflow signal; $V(t)$ is the respiratory volume signal.

S4. Respiratory controller

The equations related to the Ventilation Controller and Breathing Pattern Optimizer are the presented below. The variables include pressures (P), volumes (V) and flows ($\dot{V}$). A and D indicate alveolar and dead space compartment.

S4.1. Ventilation Controller

$$\dot{V}A = \dot{V}A_{rest} \cdot (KpCO_2 \cdot PamCO_2 + KcCO_2 \cdot PmbCO_2 + G_3 + KcMRV \cdot MRV - Kbg) \quad (S38)$$

$$G_3 = \begin{cases} KpO_2 \cdot (104 - PamO_2)^{4.9} & PamO_2 < 104 \\ 0 & PamO_2 \geq 104 \end{cases} \quad (S39)$$

$$\dot{V}A = \begin{cases} \dot{V}'A & \dot{V}'A \geq 0 \\ 0 & \dot{V}'A < 0 \end{cases} \quad (S40)$$

$$\dot{V}E = \dot{V}A + \dot{V}D \quad (S41)$$

$$\dot{V}D = BF \cdot VD \quad (S42)$$

$$VD = GV_{dead} \cdot \dot{V}A + V0_{dead} \quad (S43)$$

Where $PamO_2$, $PamCO_2$ and $PbmCO_2$ are the mean values of PaO_2, $PaCO_2$ and $PbCO_2$ in each respiratory cycle; MRV is the metabolically related neural drive component to ventilation; $\dot{V}E$ is the total minute ventilation.

Table S13. Parameters of Ventilation Controller.

Parameter	Definition
GV_{dead}	Constant gain for dead space volume
Kbg	Blood gas dissociation constant
$KcCO_2$	Constant gain of CO_2 central chemoreceptors
$KcMRV$	Constant gain of central response to exercise (neural drive)
$KpCO_2$	Constant gain of CO_2 peripheral chemoreceptors
KpO_2	Constant gain of O_2 peripheral chemoreceptors
$V0_{dead}$	Offset value of dead space volume
$\dot{V}A_{rest}$	Basal value of alveolar ventilation

S4.2. Breathing Pattern Optimizer

$$J = \dot{W}_T \quad (S44)$$

$$\dot{W}_T = \dot{W}_I + \lambda_2 \cdot \dot{W}_E \quad (S45)$$

$$\dot{W}_I = \frac{1}{(t_1 + t_2)} \int_0^{t1} \left[\frac{P_{musc}(t)}{\xi_1^n \xi_2^n} + \lambda_1 \cdot \dot{V}(t)^2 \right] dt \quad (S46)$$

$$\dot{W}_E = \frac{1}{(t_1 + t_2)} \int_{t1}^{t1+t2} \ddot{V}(t)^2 dt \quad (S47)$$

$$\xi_1 = 1 - P_{musc}(t)/P_{max} \quad (S48)$$

$$\xi_2 = 1 - \dot{P}_{musc}(t)/\dot{P}_{max} \quad (S49)$$

$$Nd = [a_0, a_1, a_2, tau, t_1, t_2] \quad (S50)$$

$$TI = t_1 \quad (S51)$$

$$BF = 1/(t_1 + t_2) \quad (S52)$$

Where J is the cost function that must be minimized and reflects the mechanical costs of breathing; TI is the inspiratory time; BF is the Breath frecuency; $\dot{W}_T$ represent the total mechanical work; $\dot{W}_I$ is mechanical work of the inspiratory phase; $\dot{W}_E$ is mechanical work of the expiratory phase; ξ_1 and ξ_2 are efficiency factors; Nd is the neural drive signal, which defines the breathing pattern subject.

Table S14. Parameters of Breathing Pattern Optimizer.

Parameter	Definition
λ_1	Weighting factor
λ_2	Weighting factor
n	Power index of efficiency factor
P_{max}	Maximum inspiratory pressure
$\dot{P}_{max}$	maximum pressure rate during inspiration

S5. Gas exchange system

The equations related to the Gas exchange system are presented below. The variables include concentrations (C), pressures (P), fractions (F), volumes (V) and flows ($\dot{V}$). Different subscripts are used: gas, the specific gas (O_2 or CO_2); dead, dead space; a, arterial; A, alveolar; v, venous.

S5.1. Gas Exchange and Mixing

$$V_{gas} \cdot \frac{dPA_{gas}}{dt} = \begin{cases} 863 \cdot (Q_{pp})(C_{v,gas} - C_{a,gas}) + \dot{V} \cdot (P_{d(5)gas} - PA_{gas}) & \dot{V}(t) \geq 0 \\ 863 \cdot (Q_{pp})(C_{v,gas} - C_{a,gas}) & \dot{V}(t) < 0 \end{cases} \quad (S53)$$

$$V_{gas} = V(t) + VL_{gas} \quad (S54)$$

$$(T1 \cdot T2) \cdot \frac{d^2Pa_{gas}}{dt^2} = PA_{gas}(t - Ta) - (T1 + T2) \cdot \frac{dPa_{gas}}{dt} - Pa_{gas} \quad (S55)$$

$$Ta = \frac{LCTV}{Q_{la}} \quad (S56)$$

$$C_{aCO_2} = (C_2 \cdot Z) \cdot \frac{F_{CO_2}{}^{1/a_2}}{1 + F_{CO_2}{}^{1/a_2}} \quad (S57)$$

$$F_{CO_2} = \frac{PACO_2(1 + \beta_2 PAO_2)}{K_2(1 + \alpha_2 PAO_2)} \quad (S58)$$

$$C_{aO_2} = (C_1 \cdot Z) \cdot \frac{F_{O_2}{}^{1/a_1}}{1 + F_{O_2}{}^{1/a_1}} \quad (S59)$$

$$F_{CO_2} = \frac{PAO_2(1+\beta_1 PACO_2)}{K_1(1+\alpha_1 PACO_2)} \quad (S60)$$

Where Q_{la} is the blood flow in the left atrium; $P_{d(5)gas}$ is the gas pressure in the fifth dead space compartment.

Table S15. Parameters of the Gas Exchange and Mixing.

Parameter	Definition
a_1	Parameter associated with the Bohr and Haldane effects regarding the O_2 dissociation in blood
a_2	Parameter associated with the Bohr and Haldane effects regarding the CO_2 dissociation in blood
α_1	Parameter associated with the Bohr and Haldane effects regarding the O_2 dissociation in blood
α_2	Parameter associated with the Bohr and Haldane effects regarding the CO_2 dissociation in blood
β_1	Parameter associated with the Bohr and Haldane effects regarding the O_2 dissociation in blood
β_2	Parameter associated with the Bohr and Haldane effects regarding the CO_2 dissociation in blood
C_1	Maximum concentration of hemoglobin-bound oxygen
C_2	Maximum carbon dioxide concentration
$P_{d(5)CO_2IC}$	Initial condition for 5th CO_2 dead space
$P_{d(5)O_2IC}$	Initial condition for 5th O_2 dead space
K_1	Parameter in O_2 dissociation equation
K_2	Parameter in CO_2 dissociation equation
$LCTV$	Lung to chemoreceptor transportation vascular volume constant
$T1$	Time constant for cardiovascular mixing
$T2$	Time constant for cardiovascular mixing
VL_{CO_2}	Lungs storage volume for CO_2
VL_{O_2}	Lungs storage volume for O_2
Z	Molar conversion factor from mmol/l to l/l

S5.2. Gas transport

Brain Compartment

$$SbCO_2 \cdot \frac{dPvbCO_2}{dt} = MRBCO_2 + Q_{bp} \cdot SCO_2(PaCO_2 - PvbCO_2) - h \quad (S61)$$

$$KCSFCO_2 \cdot \frac{dPCSFCO_2}{dt} = PvbCO_2 - PCSFCO_2 \quad (S62)$$

$$PbCO_2 = PvbCO_2 + (PCSFCO_2 - PvbCO_2) \cdot \exp^{-dc(Q_{bp} \cdot KCCO_2)^{0.5}} \quad (S63)$$

Where $PvbCO_2$ is the brain venous blood pressure of CO_2; $PCSFCO_2$ is the partial pressure of CO_2 in the cerebrospinal fluid; $PbCO_2$ is the brain carbon dioxide partial pressure (partial pressure of CO_2 at the site of central receptors).

Table S16. Parameters of of the Gas transport: Brain Compartment.

Parameter	Definition
dc	Depth of central receptor below surface of the medulla
h	Cerebral blood flow constant
$KCCO_2$	CO_2 central receptor constant
$KCSFCO_2$	CO_2 diffusion time constant of cerebrospinal fluid
$MRBCO_2$	Metabolic production rate for CO_2 in the brain tissue
$MRBO_2$	Metabolic production rate for O_2 in the brain tissue
PbCO2IC	Initial condition for brain carbon dioxide partial pressure
$SbCO_2$	Dissociation slope for CO_2 in the brain tissue
SCO_2	Dissociation slope for CO_2 in the blood

STPD is standard temperature and pressure, dry

Body Tissues Compartment

$$Q_T = Q_{pp} - Q_{bp} \quad (S64)$$

$$\tau MR \cdot \frac{dMRT_{gas}}{dt} = MR_{gas} - MRT_{gas} \quad (S65)$$

$$VTCO_2 \cdot \frac{dCvCO_2}{dt} = MRTCO_2 + Q_T(CaCO_2 - CvCO_2) \quad (S66)$$

$$VTO_2 \cdot \frac{dCvO_2}{dt} = -MRTO_2 + Q_T(CaO_2 - CvO_2) \quad (S67)$$

Where Q_T is the blood flow in the tissues; $CvCO_2$ and CvO_2 are the mixed venous CO2 and O_2 concentrations; MRT_{gas} is the metabolic tissue rate for the specific gas; MR_{gas} is the metabolic rate for the specific gas.

Table S17. Parameters of the Gas transport: Body Tissues Compartment.

Parameter	Definition
τMR	Metabolic rate time constant
$VTCO_2$	Body tissue storage volume for CO_2
VTO_2	Body tissue storage volume for O_2

S5.3. Metabolism Dynamic

$$\mathrm{M'RR} = \frac{(MRBCO_2 + MRBO_2 + \mathrm{MRTCO_2} + \mathrm{MRTO_2})}{(MRBCO_2 + MRBO_2 + MRTCO_2basal + MRTO_2basal)} \tag{S68}$$

$$\mathrm{MRR} = \begin{cases} \mathrm{M'RR} & \mathrm{M'RR} \geq 1 \\ 1 & \mathrm{M'RR} < 1 \end{cases} \tag{S69}$$

$$\tau MRV \cdot \frac{\mathrm{dM'RV}}{\mathrm{dt}} = (\mathrm{MRR} - 1) - \mathrm{M'RV} \tag{S70}$$

$$\mathrm{MRV} = \begin{cases} \mathrm{M'RV} & \mathrm{M'RV} \geq 0 \;\&\; \mathrm{MRR} > 1 \\ 0 & \mathrm{M'RV} < 0 \mid \mathrm{MRR} \leq 1 \end{cases} \tag{S71}$$

Where MRR is the metabolic rate ratio and MRV is the metabolically related neural drive component to ventilation.

Table S18. Parameters of the Gas transport: Metabolism Dynamic.

Parameter	Definition
$MRTCO_2basal$	Basal metabolic production rate for CO_2
$MRTO_2basal$	Basal metabolic consumption rate for O_2
τMRV	Metabolic rate time constant

REFERENCES

[1] C.A. Sarmiento, A.M. Hernandez, L.Y. Serna Higuita, M.Á. Mañanas, An integrated mathematical model of the cardiovascular and respiratory response to exercise: Model-building and comparison with reported models, Am. J. Physiol. Circ. Physiol. (2021) ajpheart.00074.2020. https://doi.org/10.1152/ajpheart.00074.2020.
